# Linking inter‐annual variation in environment, phenology, and abundance for a montane butterfly community

**DOI:** 10.1002/ecy.2906

**Published:** 2019-11-29

**Authors:** James E. Stewart, Javier Gutiérrez Illán, Shane A. Richards, David Gutiérrez, Robert J. Wilson

**Affiliations:** ^1^ College of Life and Environmental Sciences University of Exeter Exeter EX4 4PS UK; ^2^ Department of Entomology Washington State University Pullman Washington 99164‐6382 USA; ^3^ School of Natural Sciences University of Tasmania Hobart Tasmania 7001 Australia; ^4^ Área de Biodiversidad y Conservación Universidad Rey Juan Carlos Móstoles Madrid E28933 Spain; ^5^ Departamento de Biogeografía y Cambio Global Museo Nacional de Ciencias Naturales (MNCN‐CSIC) Madrid E28006 Spain

**Keywords:** altitude, developmental delay, ectotherm, elevation gradient, emergence time, growing season, Lepidoptera, microclimate, phenological synchrony, phenotypic traits

## Abstract

Climate change has caused widespread shifts in species’ phenology, but the consequences for population and community dynamics remain unclear because of uncertainty regarding the species‐specific drivers of phenology and abundance, and the implications for synchrony among interacting species. Here, we develop a statistical model to quantify inter‐annual variation in phenology and abundance over an environmental gradient, and use it to identify potential drivers of phenology and abundance in co‐occurring species. We fit the model to counts of 10 butterfly species with single annual generations over a mountain elevation gradient, as an exemplar system in which temporally limited availability of biotic resources and favorable abiotic conditions impose narrow windows of seasonal activity. We estimate parameters describing changes in abundance, and the peak time and duration of the flight period, over ten years (2004–2013) and across twenty sample locations (930–2,050 m) in central Spain. We also use the model outputs to investigate relationships of phenology and abundance with temperature and rainfall. Annual shifts in phenology were remarkably consistent among species, typically showing earlier flight periods during years with warm conditions in March or May–June. In contrast, inter‐annual variation in relative abundance was more variable among species, and generally less well associated with climatic conditions. Nevertheless, warmer temperatures in June were associated with increased relative population growth in three species, and five species had increased relative population growth in years with earlier flight periods. These results suggest that broadly coherent interspecific changes to phenology could help to maintain temporal synchrony in community dynamics under climate change, but that the relative composition of communities may vary due to interspecific inconsistency in population dynamic responses to climate change. However, it may still be possible to predict abundance change for species based on a robust understanding of relationships between their population dynamics and phenology, and the environmental drivers of both.

## Introduction

As the climate has warmed, the timing of life cycle events—phenology—has advanced for a wide range of taxa (Parmesan 2007). However, phenological changes have not been consistent in direction or magnitude across populations, species, or trophic levels (Thackeray et al. 2010, Scranton and Amarasekare 2018). Understanding the drivers and ecological consequences of this variation in phenology is challenging because the population sizes and activity periods of species also vary across geographical gradients, such as elevation or latitude, and because gradients in climate and habitat conditions also covary (Hodkinson 2005, Primack et al. 2009, Moussus et al. 2010). Consequently, it remains uncertain whether co‐occurring species will maintain synchrony in a changing climate (Primack et al. 2009, Bewick et al. 2016), or how changes to phenology will influence species abundance (Miller‐Rushing et al. 2010). To predict the consequences of climate change for ecological communities, we need tests of how abiotic and biotic conditions affect both the phenology and population dynamics of interacting species (Ozgul et al. 2010, Dunn and Møller 2014, McLean et al. 2016).

Phenological change could itself influence population dynamics via several mechanisms. First, if the duration of an activity period is extended, then population growth can occur over a longer period. At temperate and boreal latitudes, increased growing season length has allowed some insect species to increase the number of generations per year (Altermatt 2010a), which is expected to increase offspring production (Buckley et al. 2017). However, the population dynamic consequences of extended activity are uncertain if environmental cues for development or diapause become unreliable (Altermatt 2010a). Second, phenological shifts (advances or delays) can alter the prevailing abiotic conditions that populations are exposed to; maladaptive shifts may expose sensitive life stages to freezing or drought (Miller‐Rushing et al. 2010). They can also disrupt synchrony with food resources (Hindle et al. 2015, Posledovich et al. 2018, Renner and Zohner 2018), or with competitors and parasites (Stireman et al. 2005), affecting individual growth rate, survival and/or fecundity (Murphy et al. 1983, Posledovich et al. 2015, Fuentealba et al. 2017). These changes to synchrony could have important effects on population dynamics for species with specialized interspecific interactions, such as limited diet breadth (Altermatt 2010b), and identifying their drivers is therefore important to assess long‐term ecological effects of climate change.

The effects of phenology on the population dynamics of interacting species may be acute in mountain regions, where climatic variables change markedly over short geographic distances (Hodkinson 2005). Suitable conditions and resources may therefore be limited and/or patchy in space and time at higher elevations, particularly where they are regulated by temperature. In these regions, temperature‐sensitive species typically have narrow daily and seasonal activity periods (Gunderson and Leal 2016), narrow phenological windows for biotic interactions (Kudo and Ida 2013), and are expected to be sensitive to climate change because mountains are subject to high levels of warming (Nogués‐Bravo et al. 2007). As a result, community responses to climate change, in terms of abundance and phenology, are expected to be highly nonlinear in space and time in mountain habitats. These nonlinearities pose statistical challenges when developing models to identify and quantify the effects of climate change. For example, population densities can change markedly over elevation (Gutiérrez and Menéndez 1998, Gutiérrez Illán et al. 2010), and common measures of phenology (such as first flight date and length of flight period in insects), may be positively skewed by population size (Moussus et al. 2010). Thus, to quantify links between population dynamics and phenology reliably, we must first account for the effects of elevation and population size on measures of phenology.

In this paper, we detect responses to climate change for a montane butterfly community, using a novel statistical model that incorporates both phenology and abundance. We use the model to describe population dynamics of multiple univoltine species (having single annual generations) distributed across an environmental gradient, defined here by elevation. The model describes each species’ phenology using a Gaussian curve, defined by peak day and length of the flight period, as has been used elsewhere for the phenology of seasonal organisms (Bishop et al. 2013, Dennis et al. 2015). Importantly, the model allows interannual variations in abundance and phenology, and for phenology to vary across the gradient; such patterns are often observed in montane insect communities (Gutiérrez Illán et al. 2012). Outputs from this model can be used to quantify the degree to which interannual variation in phenology and abundance are correlated across species within a community, as well as to identify the potentially important drivers of phenology and abundance among species. Hence, the model provides a basis for estimating the expected degree of synchrony in the responses of community members to climate change, and whether synchrony among species results from common environmental drivers.

To examine the interplay between phenological shifts and population dynamics for co‐occurring species, we applied our model to 10 butterfly species that are univoltine throughout their geographic ranges in Europe, and that are therefore likely to show phenological shifts in response to climate change, rather than extended activity through increased voltinism (Altermatt 2010a, b). We analyzed count data for 10 yr (generations) from 20 sites in the Sierra de Guadarrama, a mountain range in Central Spain. Applying our model to this data set allowed us to ask (1) How do species’ phenology and abundance vary from year to year, and with elevation? We then used the model outputs to understand (2) Is there interspecific concordance in phenological and abundance change over time? (3) How do climatic conditions influence the phenology and abundance of each species? (4) How do interannual shifts in phenology (peak and duration of flight period) correlate with shifts in abundance for each species?

## Methods

### Study system

We counted butterflies along fixed transects (500 × 5 m) at 20 independent sites across an elevation gradient of 930–2,050 m in the Sierra de Guadarrama (approximately 40°45′ N, 4°00′ W). Counts were conducted every 2 wk during each annual flight season across a 10 yr period (2004–2013; Appendix S1). The data represent a series of temporally structured samples over the elevation gradient, as collection involved revisiting sites multiple times both within and between years. We focus on nonmigratory species with the most robust data: 10 species that were present in at least half of the surveyed locations in all years, with a minimum count of 35 individuals per year over all occupied sites (Appendix S1). These species are broadly ecologically similar; all are univoltine, with partially overlapping flight periods peaking between June and September. They feed on perennial grass or herbaceous host plants, and overwinter as eggs or young larvae (Settele et al. 2008).

### Climatic data

To summarize climatic conditions we obtained monthly mean temperature and precipitation data for the period 2003–2013 from the Puerto de Navacerrada weather station (AEMet 2015), which is located at 1,894 m, on average 14.0 ± 7.75 km from our study sites. Puerto de Navacerrada is the only meteorological station within the mountain range that has a full temperature and precipitation record for the study period. Data from one meteorological station occurring on the plain immediately south of the mountains (Colmenar Viejo, 1,004 m; 22.0 ± 6.1 km from the study sites) were highly correlated with those from Puerto de Navacerrada (monthly temperature variables analyzed, df = 8, *r *>* *0.94, *P *<* *0.001; quarterly rainfall data, df = 8, *r *>* *0.82, *P *<* *0.001), apart from rainfall in July to September (*r *=* *0.58, *P *=* *0.076) for which we therefore include data from both stations in our analyses. The sampling period spanned years that were climatically very different (Appendix S2: Fig. S1); for example, temperatures during March–June 2006 were 2.4°C warmer than in 2008, whilst 2005 and 2007 were particularly dry.

### Model development: patterns of phenology and abundance

We developed a statistical model (Data S1) to test for interannual changes in the timing and magnitude of adult abundance for each species across the elevation gradient. We used R version 3.4.1 or later for all analyses (R Development Core Team 2017).

In our model, we let *z*_*i*_ denote the elevation of site *i* and *d*_*i,j,y*_ the day of the year of the *j*th visit to site *i* in year *y*. The number of individuals of the focal species recorded at the site during each survey is denoted by *n*_*i,j,y*_. For the complete set of observed counts of the species (*N*), the associated days of observation (*D*), and a set of site characteristics (*X*), we seek a model that determines the probability of observing *N*, given *D* and *X*. For *X* we use elevation as the focal environmental gradient, but the method can accommodate additional covariates (e.g., plant cover and insolation) for cases in which the sample size allows for estimation of additional parameters.

First, we consider the probability of observing butterfly abundance of a focal species at a specific site. The expected number of butterflies observed at site *i* on day *d* in year *y* is denoted ${\bar{n}}_{\left(i,y,d\right)},$ and is assumed to be composed of three parts:

(1) $${\bar{n}}_{\left(i,y,d\right)}=N_{\left(i,y\right)}T_{\left(i,y,d\right)}.$$

*T*_(*i*,*y*,*d*)_ describes the phenology of the species and is bounded between 0 and 1. *N*_(*i*,*y*)_ describes the maximum expected number of butterflies at site *i* during year *y*, and is assumed to have the following form:

(2) $$N_{\left(i,y\right)}=n_{i}^{*}\exp\left(U_{y}\right)$$

where $n_{i}^{*}$ is the most likely peak butterfly count across all years for site *i*. *U*_*y*_ allows for temporal (interannual) variation in abundance, describing how abundance across all sites is affected by unmeasured large‐scale variables (e.g., climatic effects) during year *y*. *U*_*y*_ sums to 0 over the 10 yr.

Butterfly flight period (phenology) was modeled using a Gaussian curve, which is common in models of univoltine butterfly phenology (Bishop et al. 2013, Dennis et al. 2015) and representative of the 10 species observed here (Appendix S2: Figs. S2–S11):

(3) $$T_{\left(i,y,d\right)}=\exp\left(-\frac{1}{2}{\left(\frac{d-{\bar{d}}_{i,y}-Q_{y}}{s_{d,i,y}}\right)}^{2}\right)$$

where

(4) $${\bar{d}}_{i,y}=d^{*}\left(1+h_{1}z_{i}+h_{2}z_{i}^{2}\right)$$

is the expected peak day of observation at site *i* in year *y*. Here, peak day at the mean elevation of the study sites is d* days, and we have allowed phenology to vary along the elevational gradient: the peak day may have either a constant, linear (*h*_1_), or quadratic (*h*_2_) relation with normalized site elevation (*z*_*i*_). Potential limits to phenological change with elevation may be captured by the quadratic term. The term,

(5) $$s_{d,i,y}=s_{d}^{*}\left(1+gz_{i}\right)\exp\left(R_{y}\right)$$

is the standard deviation of the phenology period at site *i* in year *y*. Flight period for sites at the mean elevation is defined by $s_{d}^{*}$, and flight period may have a linear relation with elevation (*g*). Interannual variation in both the peak day, and the duration of the flight period, across all sites, are described by *Q*_*y*_ and *R*_*y*_, respectively. As with *U*_*y*_, both of these sets of model parameters sum to 0. Specifically, *Q*_*y*_ describes the number of days the flight period is shifted later in the year in year *y*, and *R*_*y*_ describes relative change in the duration of the flight period in year *y*.

Table 1 summarizes all parameters in Eqs. (1), (2), (3), (4), (5), which provide the expected count of a single species at a site. To determine the probability of observing a specific count we assume that counts follow a negative‐binomial distribution (NBD), which is common in ecology (Richards 2008). The probability of observing all the data is

(6) $$\mathrm{Pr}\left(N|D,X,\theta \right)=\prod_{i=1}^{20}\prod_{j=1}^{J(i,y)}\prod_{y=2004}^{2013}\text{NBD}\left(n_{i,j,y};\bar{n}\left(i,d_{i,j,y},y\right),\phi \right)$$

where *J*(*i*,*y*) is the number of times site *i* was visited in year *y*, and θ is the set of parameters needed to compute $\bar{n}$. Here, the NBD is parameterized according to

(7) $$\text{NBD}\left(n;\bar{n},\phi \right)=\frac{\ln\Gamma (n+a)}{\Gamma (n+1)\Gamma (a)}{\left(\frac{b}{1+b}\right)}^{a}{\left(1+b\right)}^{-n}$$

where Γ(.) is the complete gamma function, $a=\bar{n}/\phi$ and *b *=* *1/ϕ. $\text{NBD}(n;\bar{n},\phi )$ is the probability of observing count *n* when the expected count is $\bar{n}$ and ϕ is the overdispersion parameter describing variation among observed counts, such that the variance in counts is $(1+\phi )\bar{n}$.

**Table 1:** Summary of model parameters

Parameter	Description
$n_{i}^{*}$	Maximum expected count at peak time at site *i*
d*	Day of year on which abundance peaks
*s*_*d*_	Standard deviation of the width of the phenology period (days)
ϕ	Overdispersion parameter describing variation among observed counts
*h*_1_, *h*_2_ †	Linear and quadratic terms relating peak day with elevation
*g* †	Linear term relating elevation with width of the phenology period
*Q*_*y*_ †	Yearly effect on peak timing‡
*R*_*y*_ †	Yearly effect on width of the emergence period§
*U*_*y*_ †	Yearly effect on peak abundance¶

Note

For *Q*_*y*_ *, R*_*y*_, and *U*_*y*_ *, y *=* *2004–2013.

† Parameters that may be set to zero, thereby removing the effect of the associated variable; it is possible to run the model without these parameters, but their inclusion allows specific yearly changes or effects of elevation to be tested for.

‡ Represents the phenological shift in year *y* relative to the 2004–2013 average.

§ Represents the duration of emergence in year *y* relative to the 2004–2013 average.

¶ Represents the species’ abundance in year *y* relative to the 2004–2013 average. From this, we calculate $\rho_{y}$, which describes the relative abundance change from one year to the next.

### Model selection

The first four parameters in Table 1 must be specified to model the probability of observing each count. A set of more specific models was constructed by allowing unique combinations of the remaining parameters to differ from zero. For example, yearly effects occur whenever one or more of the parameter groupings *Q*_*y*_, *R*_*y*_, or *U*_*y*_ are nonzero. We constructed a candidate set of models by considering all possible parameter combinations, and used the R package Rvmmin (Nash 2017) to calculate the maximum‐likelihood parameter estimates. Akaike's Information Criterion–based model selection was used to determine which of the models were most parsimonious with respect to the data (Appendix S1). Models were retained in a candidate set if they had an AIC value within six of the minimum AIC calculated and there was no simpler model with a lower AIC (Richards 2008). For the instances in which this process failed to identify a single “best” model, we base biological inference on the simplest model (i.e., the model with the fewest parameters), and consider a parameter to have strong support if it is included (i.e., was nonzero) in all candidate models (Richards 2015; Appendix S2: Table S1).

### Variation in phenology, abundance, and climatic conditions

Changes in phenology, *Q*_*y*_ (day of peak abundance, hereafter “peak timing”) and *R*_*y*_ (duration of emergence), are reported for each year *y* relative to the average for the 10 yr period 2004–2013. Changes in peak abundance *U*_*y*_ are reported in a modified form as $\rho_{y}$, letting

(8) $$\rho_{y}=\exp\left(U_{y}-U_{y-1}\right)$$

in this way, the reported $\rho_{y}$ account for abundance in the previous year. As no population data were available for 2003, it was not possible to calculate ρ_2004_; ρ_*y*_ are therefore reported for 2005–2013. Spearman's rank correlation was used to identify associations between *Q*_*y*_ and $\rho_{y}$. Interspecific concordance in phenological and abundance change was calculated as the proportion of species whose direction of change matched the community average direction of change for a given year.

Generalized linear models were used to look for evidence that climatic conditions influenced relative abundance ($\rho_{y}$) and phenology (*Q*_*y*_) of a given species. We considered monthly temperatures from January to June of year *y*, and quarterly rainfall between July of year *y *− 1 to June of year *y*. We use a longer time frame for rainfall data to capture potential previous‐year effects of rainfall on host plant condition. We limited all GLMs to a maximum of three predictors per species due to low sample sizes ($\rho_{y}$, *n* = 9; *Q*_*y*_, *n* = 10). We used AIC‐based model selection (above) to define the most parsimonious model. All GLMs used Gaussian errors with identity link except for cases in which model diagnostics and fit were improved by use of an inverse link. We assessed variance inflation factors and found a lack of significant collinearity amongst predictors in all final models (Zuur et al. 2010). This method identified environmental drivers of phenology that appeared to be common to all of our 10 species; we then used these common drivers in a general linear model to estimate community phenology responses to warming, using AIC to compare this with a null model.

## Results

### How do phenology and abundance vary over elevation and time?

**Figure 1:**
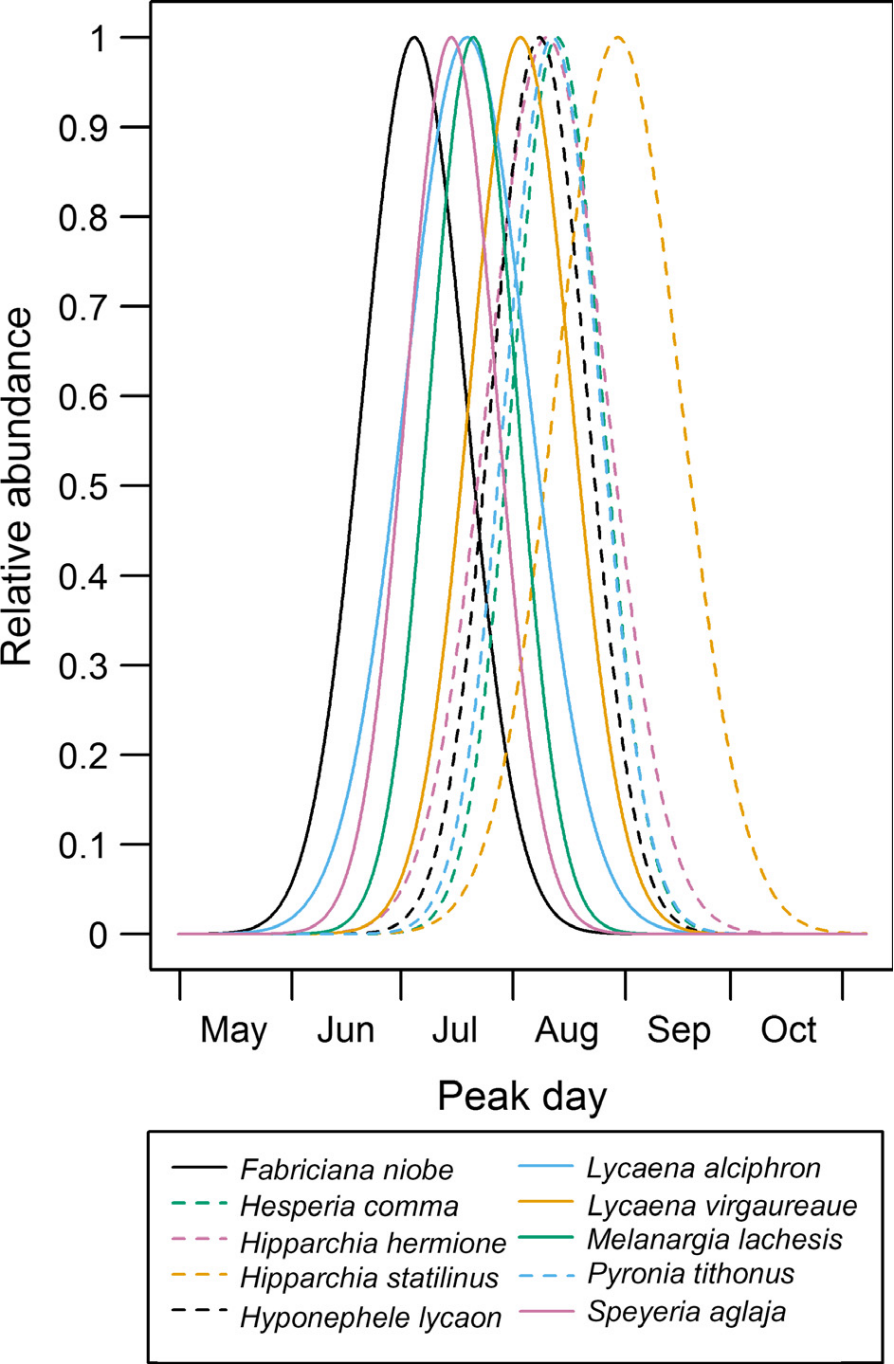
Predicted flight period timing for 10 butterfly species, shown in terms of relative abundance (as a proportion of total abundance over the season). Predictions are presented for the best‐fit model (Table 2) and are based on the parameters d* and *s*_*d*_ (Table 1).

There was marked interspecific variation in timing of emergence (Fig. 1; Table 2). Peak timing varied between species by up to 56 d (d*; range 185–241) and the standard deviation around this (*s*_*d*_) varied between 12 and 17 d. Assuming Gaussian variation in emergence, approximately 95% of conspecifics of a species will be present as adults between the peak emergence time, d*, plus or minus two *s*_*d*_; therefore, the flight periods of these species ranged between approximately 48 and 69 d (Fig. 1).

**Table 2:** Summary of the output from the selected phenology model for 10 butterfly species, presenting key parameter values, best‐fitting functional forms, and evidence of elevation and yearly effects

Description	Parameter/function	Species									
		*Fabriciana niobe*	*Hesperia comma*	*Hipparchia hermione*	*Hipparchia statilinus*	*Hyponephele lycaon*	*Lycaena alciphron*	*Lycaena virgaureaue*	*Melanargia lachesis*	*Pyronia tithonus*	*Speyeria aglaja*
*n* observed	${\bar{n}}_{i,j,y}$	1,323	1,967	1,400	1,850	3,700	703	4,904	14,569	4,489	1,266
Baseline parameters											
Peak abundance†	$n_{i}^{*}$										
Peak day	d*	185.2	224.6	221.8	241.4	219.7	199.8	214.5	201.5	223.4	195.4
SD of peak day	*s*_*d*_	14.2	12.3	16.4	17.2	13.1	17.1	14.0	11.9	12.9	12.4
Variation in counts	$(1+\phi )\bar{n}$	1.36	1.55	0.60	0.53	1.94	0.24	2.01	2.00	1.98	0.71
Elevation effects											
Peak day	$d^{*}\left(1+h_{1}z_{i}+h_{2}z_{i}^{2}\right)$	Quadratic	Linear	Constant	Linear	Quadratic	Quadratic	Quadratic	Linear	Linear	Linear
Linear	*h*_1_	0.0287	0.0254	–	0.0163	0.0315	0.0368	0.0371	0.0363	0.0352	0.0535
Quadratic	*h*_2_	0.0130	–	–	–	−0.0158	−0.0247	−0.0237	–	–	–
SD of duration	$s_{d}^{*}\left(1+gz_{i}\right)$	Constant	Constant	Constant	Constant	Constant	Constant	Constant	Constant	Constant	Constant
*g*		–	–	–	–	–	–	–	–	–	
Yearly effects											
Peak day	*Q*_*y*_	Yes	Yes	Yes	Yes	Yes	Yes	Yes	Yes	Yes	Yes
Duration	*R*_*y*_	No	Yes	Yes	Yes	Yes	No	No	Yes	No	No
Abundance	*U*_*y*_	Yes	Yes	No	Yes	Yes	Yes	Yes	Yes	Yes	Yes

Note

See main text and Appendix S2: Table S1 for model selection information.

† Unique values for each species:site combination. See Appendix S2: Table S2 for details.

All species except *Hipparchia hermione* emerged later at higher elevations (*h*_1_ and *h*_2_, Table 2). There was a simple linear relationship between elevation and phenology (*h*_1_ was nonzero) for five species, and four others showed a more complex quadratic relationship (*h*_2_ was also nonzero). Three of these species (*Hyponephele lycaon*,* Lycaena alciphron*, and *Lycaena virgaureae*) showed a slight negative quadratic effect of elevation on phenology, indicating a plateau at a maximum phenological delay above a threshold elevation (approximately 1,650 m), whereas *Fabriciana niobe* had a positive quadratic effect of elevation on phenology. The length of all species’ flight periods appeared to be the same regardless of elevation (*g*; Table 2).

All 10 species experienced interannual variation in peak timing (*Q*_*y*_; Table 2), and only one (*H. hermione*) showed no interannual variation in the magnitude of peak abundance (*U*_*y*_; Table 2). Half of the 10 species showed interannual change in the duration of the flight period (*R*_*y*_; Table 2); we therefore focus on *Q*_*y*_ as our measure of phenological change.

### Is there interspecific concordance in phenological and abundance changes?

**Figure 2:**
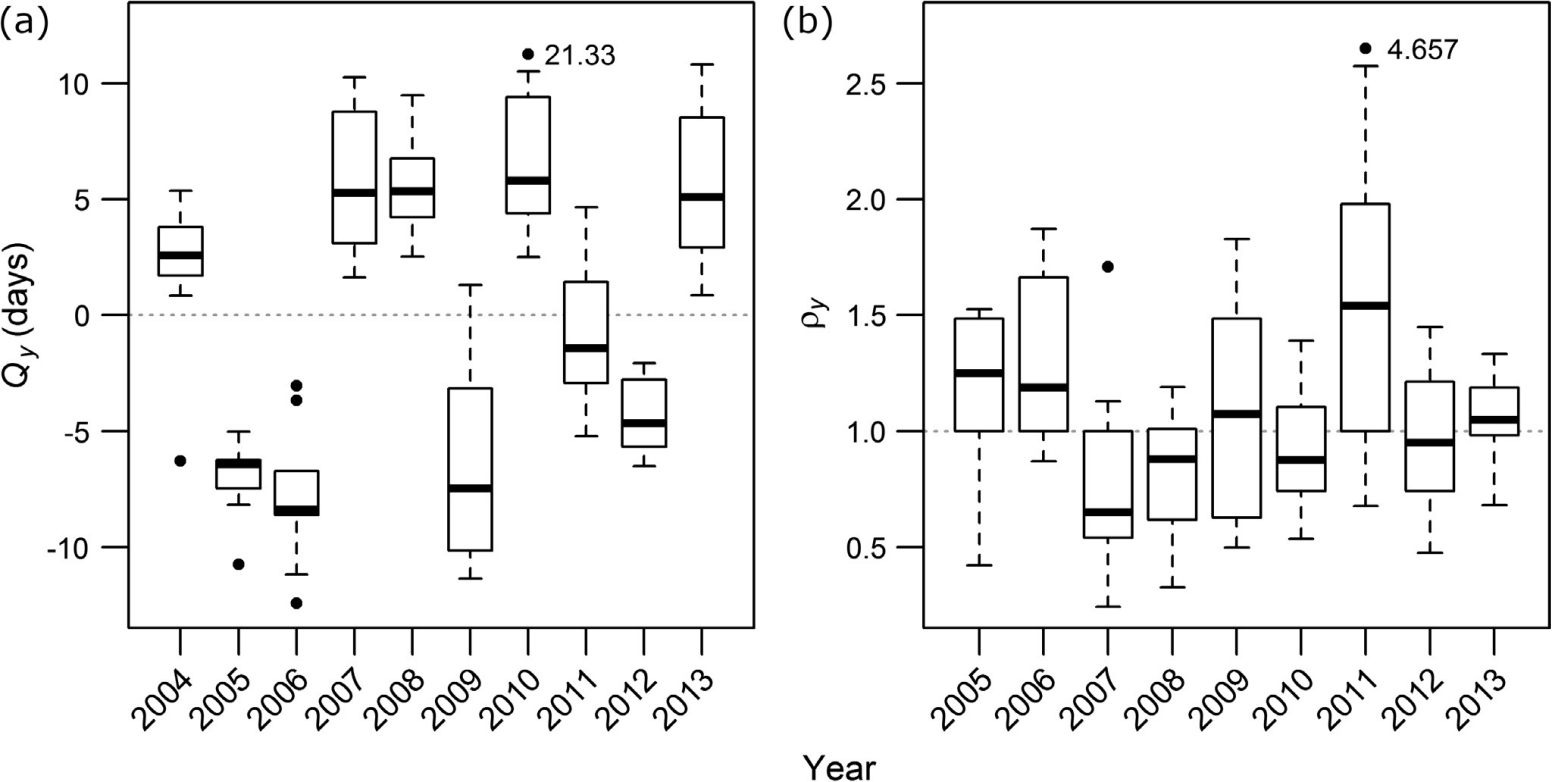
Interannual changes in (a) phenology relative to the 2004–2013 average (*Q*_*y*_ *;* days), and (b) abundance relative to the previous year ($\rho_{y}$; proportional), summarizing data for all species. Where outlier position has been altered for clarity, the numerical value of the outlier is indicated on the plot. Horizontal dotted lines indicate no change relative to average peak timing (a) or abundance in the previous year (b).

In most years, the direction of phenological shift was consistent across species (Fig. 2a). For example, years with warmer springs (such as 2005 and 2006, Appendix S2: Fig. S1) were associated with earlier adult emergence in all species, which showed a similar rate of phenological advancement. Annual abundance changes were less consistent across species (Fig. 2b): in any given year some species showed population growth ($\rho_{y}$ > 1), whereas others experienced decline ($\rho_{y}$ < 1). Overall, there was only 62% concordance in the direction of population growth, contrasted with 95% concordance in phenological shifts among species.

### How do climatic conditions determine patterns of phenology and abundance?

**Figure 3:**
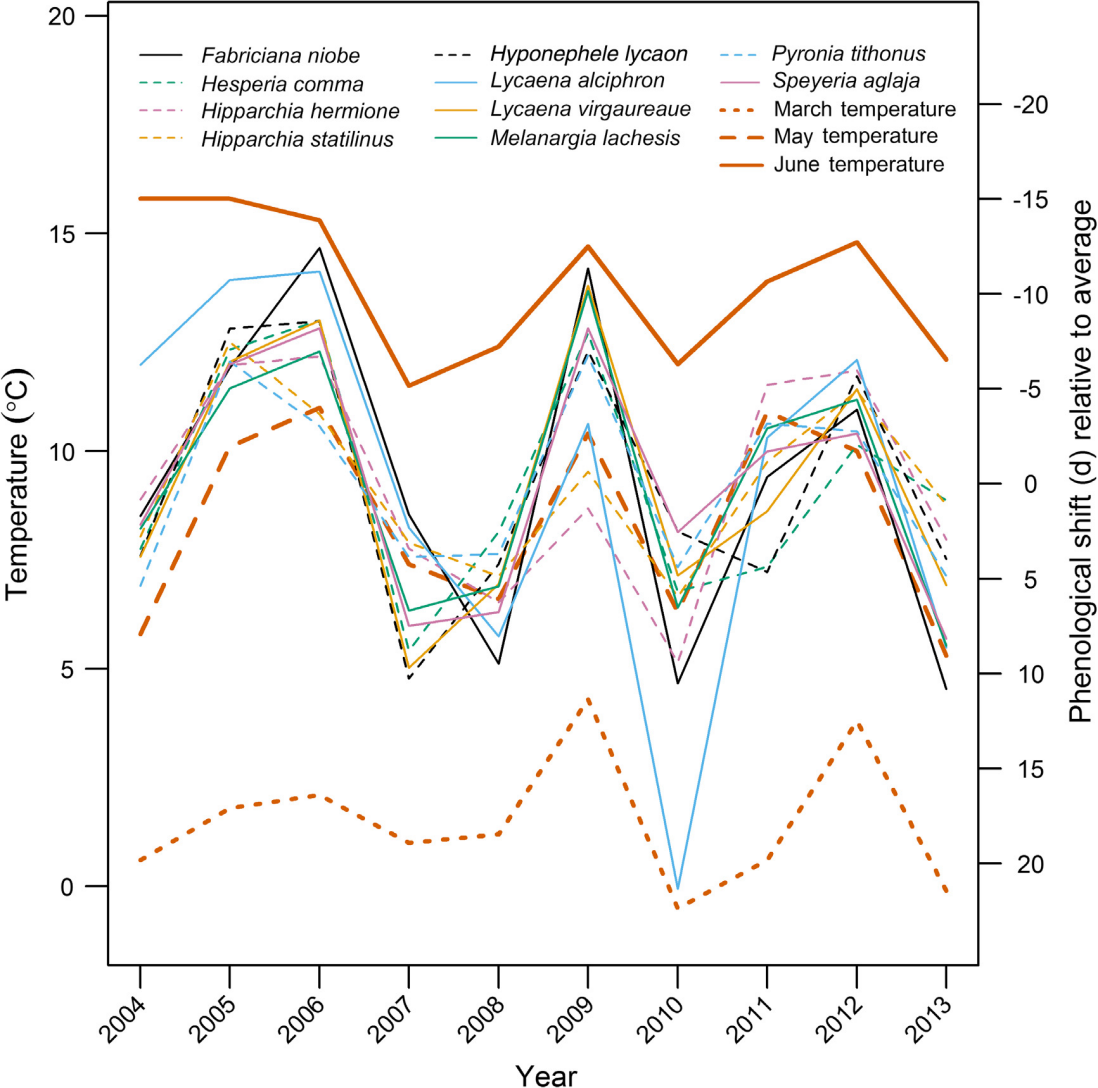
Phenological shift (*Q*_*y*_; days, relative to the 2004–2013 average peak day) varies in line with March and May–June temperatures, as per Table 3.

Phenological changes were typically driven by temperature in March and/or May–June of the current year, with early peak timing associated with high temperatures in these months, and little impact of rainfall (Fig. 3, Table 3a; Appendix S2: Table S3). A GLM of annual phenological shift (*Q*_*y*_, averaged across species) against mean March–June temperature performed better than a null model (ΔAIC = −13.0) and indicates that, for each 1°C of warming, these species peak 4.4 (±0.84, *n* = 10) days earlier. The dominant climatic correlates of year‐to‐year species abundance changes were less consistent across the 10 species than those of phenological change (Table 3; Appendix S2: Table S4), though the abundance of three species increased under higher June temperatures. We also identified dominant effects of rainfall from as early as the summer of year *y *− 1, and limited evidence for further effects of summer rainfall (Appendix S2: Table S4). However, we failed to identify climatic correlates of abundance for five species (Table 3; Appendix S2: Table S4).

**Table 3:** Rainfall and temperature effects on phenology (peak day, *Q*_*y*_; a) and abundance ($\rho_{y}$; b)

Species	β_0_	*R*_1a_	*R*_1b_	*R*_2_	*R*_3_	*R*_4_	*T*_Jan_	*T*_Feb_	*T*_Mar_	*T*_Apr_	*T*_May_	*T*_Jun_
(a) Rainfall and temperature effects on phenology (*Q*_*y*_)												
*Fabriciana niobe*	0	0	0	0	0	0	0	0	0	0	**−7.32**	0
*Hesperia comma*	0	0	0	0	0	0	0	0	0	0	0	**−4.26**
*Hipparchia hermione*	0	0	0	0	0	0	0	0	0	0	**−4.53**	0
*Hipparchia statilinus*	0	0	0	0	0	3.18	−3.04	0	0	0	**−3.41**	0
*Hyponephele lycaon*	0	0	3.42	0	0	0	0	1.51	0	0	0	**−5.47**
*Lycaena alciphron*	0	0	0	−4.02	0	0	0	0	0	0	**−7.06**	0
*Lycaena virgaureae*	0	0	0	0	0	0	2.76	0	0	3.25	**−5.22**	0
*Melanargia lachesis*	0	0	0	0	0	0	0	0	−**5.38**	−3.06	0	−3.22
*Pyronia tithonus*	0	0	0	0	0	0	0	0	0	0.85	**−4.66**	0
*Speyeria aglaja*	0	0	0	0	0	0	2.30	0	0	0	**−4.73**	0
(b) Rainfall and temperature effects on abundance ($\rho_{y}$)												
*F. niobe*	1.19	–	–	–	–	–	–	–	–	–	–	–
H. comma*	1.06	0	0	0	0	0	−0.08	0	0	−0.10	0	**−0.30**
*H. hermione* †												
*H. statilinus*	1.00	–	–	–	–	–	–	–	–	–	–	–
*H. lycaon*	1.05	0	0	0.39	0	0	0	0	0	0	−0.55	**0.69**
*L. alciphron*	1.10	–	–	–	–	–	–	–	–	–	–	–
L. virgaureae*	0.99	0	0	0	0	0	0	0	0	0	0	**−0.19**
*M. lachesis*	1.06	–	–	–	–	–	–	–	–	–	–	–
P. tithonus*	1.09	0	**−0.32**	0	0	−0.22	0	0	0	0	0	0
*S. aglaja*	1.12	–	–	–	–	–	–	–	–	–	–	–

Notes

Predictors were z‐transformed so the regression coefficients represent relative effect sizes, the largest of which is presented in bold for each species; where the null model was the most parsimonious, this is indicated with ‘–’ in place of all coefficient estimates except β_0_. All results are derived from a GLM with Gaussian error structure and identity link, except where use of an inverse link is indicated by *. For models with an inverse link, the sign of the regression coefficients is reversed, such that a negative coefficient reported here is indicative of a positive effect of that variable. See Appendix S2: Tables S3, S4 for full AIC model selection results. Combinations of up to three predictors were considered from the following: rain in July–September (*R*_1a_) and October–December (*R*_2_) of year *y *− 1, rain in January–March (*R*_3_) and April–June (*R*_4_) of year *y* and monthly temperatures of January–June in year *y* (*T*_Jan_–*T*_Jun_) at the primary weather station, in addition to rain in July–September at the secondary weather station, Colmenar Viejo (R1b).

† No yearly abundance effects were detected for *H. hermione*.

### Do shifts in phenology correlate with shifts in abundance?

Five species showed significant relationships between phenology (annual change in peak timing; *Q*_*y*_) and population dynamics (abundance relative to the previous year, $\rho_{y}$) (Table 4). In these five species, abundance was significantly higher—relative to the previous year—in years with earlier phenology.

**Table 4:** Spearman's rank correlation between annual change in peak timing (*Q*_*y*_) and annual proportional change in abundance ($\rho_{y}$) for nine species between 2005 and 2013

*Species*	Spearman's rho	*P*
*Fabriciana niobe*	−0.10	0.810
*Hesperia comma*	−0.35	0.359
*Hipparchia statilinus*	0.30	0.795
***Hyponephele lycaon***	**−0.75**	**0.025**
***Lycaena alciphron***	**−0.78**	**0.017**
***Lycaena virgaureae***	**−0.72**	**0.036**
***Melanargia lachesis***	**−0.78**	**0.017**
*Pyronia tithonus*	0.38	0.313
***Speyeria aglaja***	**−0.73**	**0.031**

Notes

The best‐fit model for the tenth species, *Hipparchia hermione*, did not include terms for annual changes in abundance. All significant correlations (bold) are negative correlations, suggesting larger proportional increases in abundance in years when the focal species emerges earlier at our 20 sites. *n* = 9 in all cases.

## Discussion

Most studies on the responses of animal taxa to climate change focus on lower‐level responses such as phenology, physiology, behavior, and demographic rates (Glanville and Seebacher 2006, Leech and Crick 2007, Sherry et al. 2007, Ozgul et al. 2010), and do not explicitly consider the consequences for population size (McLean et al. 2016). Here, we develop an approach that can detect relationships between phenology and abundance, and is adaptable to other study systems. We demonstrate how our model can be applied to temporally structured data spanning environmental gradients, as are common in long‐term ecological studies. In our case, we found that interannual changes in phenology were considerably more consistent across species than were changes in abundance and, for five species, advances in phenology (earlier peak timing) were associated with positive abundance change. The results suggest common phenological responses to climatic variation across species, but that the climatic drivers and outcomes of abundance change may be less coherent among co‐occurring species.

### Phenological variation in space and time

In our study, phenological variation across years was very consistent among the 10 species, implying common phenological drivers. In general, emergence was earlier in warm years (Fig. 3, Appendix S2: Fig. S1; Table 3), and peak abundance was observed 4.4 d earlier for each 1°C of warming, which is consistent with shifts estimated more widely for insect pollinators (4 d/°C over the last century; Memmott et al. 2007). For all species we found effects on phenology of temperature in the current year, with high temperatures between March and June associated with early emergence (Table 3a). This result suggests a link between temperature during larval (March–May) or pupal (May–June) phases and the timing of adult emergence, and is consistent with research showing advanced insect phenology following warm springs (Stefanescu et al. 2003).

The phenology of all species except one was also delayed at higher elevations (see also Gutiérrez Illán et al. 2012), further supporting a role of temperature in driving phenology. For three species there was evidence of a plateau in peak flight date at the end of the season at high elevations. At the limits of environmental gradients, populations can encounter climatic limits to behavioral or phenological plasticity (Kingsolver and Buckley 2018), limited activity periods (Gutiérrez Illán et al. 2012), or increasingly restricted availability of resources (Gutiérrez et al. 2016). However, populations toward environmental range limits may also exploit microclimates that are more similar to ambient conditions experienced closer to the core of the range, and that therefore generate similar phenology (Hindle et al. 2015).

### Linking phenology and abundance change

Whereas phenological changes were consistent across species, there was little interspecific concordance in annual variation in abundance (Fig. 2). Whilst phenology appeared to depend fundamentally on spring–summer temperatures, abundance change appeared to be less sensitive to climatic drivers. We identified associations between climate and abundance in only 4 out of 10 species, 3 of which were more abundant following higher June temperatures (Table 3b). There was also evidence for effects of rainfall in the preceding and current summers (Appendix S2: Table S4). This apparent lack of consistent climatic drivers across the 10 species may help to explain why interannual abundance change was less consistent than interannual phenological change.

Nevertheless, we found that years of early emergence were associated with increases in abundance in five species (Table 4). Flight was earlier for all of these species when spring–summer temperatures were high, and abundance was greater under warm summer conditions for two of these species. These results are consistent with favorable spring–summer conditions promoting faster development rates in temperate insects, provided that other resources are not limiting (Roy et al. 2001, Bale et al. 2002, Schenk et al. 2018). Our results suggest a link between phenological change and interannual variation in abundance, which, in some cases but seemingly not all, may be explained by common environmental drivers of these phenomena.

### Further applications and concluding remarks

Our model quantified the phenology and abundance of 10 univoltine species, detected evidence of their interannual variations, and identified how phenology varies with elevation. Although we were unable to do so with these data (Appendix S1), future studies considering larger sample sizes could extend the approach to consider the impact of additional covariates, as outlined in the R code provided (Data S1). For example, sites may differ in insolation or vegetation cover (and therefore microclimate), which could modify phenology or abundance responses to climate change or along a focal environmental gradient (Gutiérrez Illán et al. 2010, Hindle et al. 2015). Quantifying these fine‐scale differences between sites could allow a better understanding of the drivers of phenology and abundance changes, and provide insight into potential habitat management strategies (Brambilla et al. 2018).

Overall, our results demonstrate interspecific consistency in phenological responses to interannual climatic variation, but substantial interspecific variability in numerical population responses. On the one hand, our results suggest that—because of common environmental drivers—temporal synchrony among some co‐occurring species could be largely maintained in a changing climate, and that understanding phenological change (or the potential disruption of phenological synchrony), may not always be required for understanding changes to the abundance of individual species. Instead, if approaches such as ours can be used to identify species’ abundance changes and their correlates, then these relationships might themselves hold for predicting population dynamic responses in a changing climate.

On the other hand, the lack of consistent environmental drivers of abundance across 10 ecologically similar species, and the variety of phenology‐abundance relationships, highlights an inherent difficulty in predicting population dynamic impacts of future climatic change. For cases in which demographic data are available, the links between phenology and abundance may be explored in the hierarchical decomposition framework of McLean et al. (2016) to determine how, and in which circumstances, changes in climate and in traits (such as phenology) do translate to changes in abundance. To establish the generality of our conclusions, we encourage tests of the drivers of both phenology and abundance over realistic environmental gradients, for a range of co‐occurring taxa and functional groups.

## Data Availability

Data are available from ORE: Open Research Exeter at https://doi.org/10.24378/exe.1963.
