# Supplementary material for: Linking inter‐annual variation in environment, phenology, and abundance for a montane butterfly community
Source: Ecology. 2019 Nov 29;101(1):e02906. doi: 10.1002/ecy.2906 (PMC9285533; doi:10.1002/ecy.2906)
Supplement: Supplementary file 1 [file ECY-101-e02906-s003.pdf]

Supporting Information. James E. Stewart, Javier Gutiérrez Illán, Shane A. Richards, David Gutiérrez, and Robert J. Wilson. 2019. Linking inter-annual variation in environment, phenology, and abundance for a montane butterfly community. *Ecology*.

Appendix S1: Supplementary Methods

Section S1: Study system and data collection

We sampled butterflies at 20 sites in the Sierra de Guadarrama, a 100 × 30 km mountain range in central Spain that reaches a maximum elevation of 2428 metres above sea level, rising from plains to the north and south at ≥700 and ≥500 m elevation, respectively. The sites were selected based on accessibility and to provide a representative sample of the elevation gradient, ranging from 900–2050 m, with a mean of 1456 m and standard deviation of 373 m. Typical vegetation types vary with elevation, from evergreen broadleaf woodland below 1000 m to deciduous woodland at 1000–1500 m and coniferous woodland at roughly 1500–2000 m. All sample sites consisted of scrub or open grassland areas in natural or semi-natural habitat. For the species analyzed here, all sites are considered to represent independent populations in which abundance is likely to be driven by local conditions rather than by immigration from neighboring sites: nearest neighboring sites were 3.35 ± 0.65 (SE) km apart.

On the first visit to each site we established a 500 m transect route and recorded the Universal Transverse Mercator (UTM) coordinates to the nearest 1 m at least every 100 m along the route, using a handheld Garmin GPS unit. We plotted transect routes in ArcMap 10.2.2 (ESRI 2014), and overlaid them on a 5 m resolution digital elevation model (IGN 2015), subsequently

calculating the mean elevation of each transect based on the average elevation of all 5 m grid cells within a buffer zone (25 m radius) around the linear route of the transect.

Data collection

Butterfly data

We counted butterflies along a standardized transect (500 m long × 5 m wide) at each site. We use counts taken at two-week intervals between April and mid-October during suitable conditions for butterfly activity (Pollard and Yates 1993). We walked transects over ten successive years (2004–2013), with the number of counts (dependent on first count date) increasing over time to ensure that the entire flight period of each species was observed (2004, $n = 11$; 2005–2009, $n = 12$; 2010–2013, $n = 13$). In practice, the earliest observation of any species in our analyses was 16 days after the annual start date of the first surveys, so it is unlikely that individuals were missed in the years with fewer surveys.

Species included in analysis

The ten focal species included five Satyrinae (*Hipparchia hermione*, *Hipparchia statilinus*, *Hyponephele lycaon*, *Melanargia lachesis* and *Pyronia tithonus*), two Heliconiinae *(Fabriciana niobe* and *Speyeria aglaja*), two Lycaeniinae (*Lycaena alciphron* and *Lycaena virgaureae*) and one Hesperiinae species (*Hesperia comma*). Our nomenclature follows the taxonomy of Settele et al. (2008) and Wiemers et al. (2018), accounting for the recent reorganization of *Argynnis* species into three genera: *Argynnis*, *Fabriciana* and *Speyeria* (de Moya et al. 2017).

Section S2: Statistical model of phenology and abundance

Our modeling approach is generalizable to repeated count data of other taxa found along an environmental gradient, elevation or otherwise. Furthermore, it is possible to extend the approach to include relevant site-specific abiotic variables such as aspect, insolation and habitat cover. However, in attempting to fit such models we found that, in this case, the inclusion of these parameters led to unreliability in model convergence and fitting; to fit models with these additional parameters, more data would be required than are available here.

Model selection

For each of the ten butterfly species we sought a candidate set of models that were consistent with the data, and used Akaike's Information Criterion (AIC) as our model selection criterion. The AIC value associated with model M, which is defined by the set of non-zero parameters $\boldsymbol{\theta}_M$, is given by

$$\mathrm{AIC(M)} = -2\ln L\left(\theta_M|\mathrm{N,D,X}\right) + 2K_M, \quad \text{(Eq. S1)}$$

where K_M is the number of estimated model parameters (i.e., the number of parameters in the set $\boldsymbol{\theta}_M$) and $L(\boldsymbol{\theta}_M|\mathbf{N},\mathbf{D},\mathbf{X})$ is the likelihood of the model given the data, which is equal to $\Pr(\mathbf{N}|\mathbf{D},\mathbf{X},\boldsymbol{\theta}_M)$ (see Equation 6, main text).

The model having the lowest AIC value is likely the most parsimonious; however, as AIC is only an estimate of parsimony, we followed Richards (2008) in considering certain other models as well. First, we determined which models generated AIC values within 6 units of the model having the lowest AIC value. We used Δ(M) to denote the difference between the AIC value of model M and the lowest AIC value calculated. Thus, the best AIC model had $\Delta(\mathrm{M}) = 0$ and all selected models had a Δ(M)-value ≤ 6. Next, to prevent selecting unsupported, overly-complex

models, we removed models from the candidate set that were more complex versions of other selected models (Richards 2008). For example, if allowing *g* to be non-zero did not result in a lower Δ(M)-value, then we did not consider there to be evidence that site elevation affected the duration of the emergence period. For any instances in which this process failed to identify a single 'best' model, we follow Richards (2015) in basing biological inference on the simplest (lowest *k*) model (or the best AIC model if *k* is equal across candidate models) and considering a parameter to have strong support if it is included in all candidate models.

Literature Cited

de Moya, R.S., Savage, W.K., Tenney, C., Bao, X., Wahlberg, N. and Hill, R.I. (2017). Interrelationships and diversification of *Argynnis* Fabricius and *Speyeria* Scudder butterflies. *Systematic Entomology*, **42**: 635–649.

ESRI (2014). ArcGIS Desktop: Release 10.2.2. Redlands, CA, USA: Environmental Systems Research Institute.

Instituto Geográfico Nacional (2015). MDT05-LIDAR Digital Elevation Model. PNOA assigned by © Instituto Geográfico Nacional, Madrid, Spain.

Pollard, E. and Yates, T.J. (1993). *Monitoring Butterflies for Ecology and Conservation*. Chapman and Hall, London, U.K.

Richards, S.A. (2008). Dealing with overdispersed count data in applied ecology. *Journal of Applied Ecology*, **45:** 218–227.

Richards, S.A. (2015). Likelihood and model selection; pp. 58–80 *in*: Fox, G.A., Negrete-Yankelevich, S. and Sosa, V.J. (eds.) (2015). *Ecological Statistics: Contemporary Theory and Application*. Oxford University Press, Oxford, United Kingdom, 389 pp.

Settele, J., Kudrna, O., Harpke, A., Kühn, I., van Swaay, C., Verovnik, R., Warren, M., Wiemers, M., Hanspach, J., Hickler, T., Kühn, E., van Halder, I., Veling, K., Vliegenthart, A., Wynhoff, I. and Schweiger, O. (2008). Climatic Risk Atlas of European Butterflies. *BioRisk*, **1**: 1–712.

Wiemers, M., Balleto, E., Dincă, V., Faltynek, F.Z., Lamas, G., Lukhtanov, V., Munguira, M.L., van Swaay, C.A.M., Vila, R., Vliegenhart, A., Wahlberg, N., Verovnik, R. (2018). An updated checklist of the European Butterflies (Leipdoptera, Papilionoidea). *Zookeys*, 811: 9–45.
