# Supplementary material for: Linking inter‐annual variation in environment, phenology, and abundance for a montane butterfly community
Source: Ecology. 2019 Nov 29;101(1):e02906. doi: 10.1002/ecy.2906 (PMC9285533; doi:10.1002/ecy.2906)
Supplement: Supplementary file 2 [file ECY-101-e02906-s002.pdf]

Supporting Information. James E. Stewart, Javier Gutiérrez Illán, Shane A. Richards, David Gutiérrez, and Robert J. Wilson. 2019. Linking inter-annual variation in environment, phenology, and abundance for a montane butterfly community. *Ecology*.

Appendix S2: Supplementary Figures and Tables

Figure S1

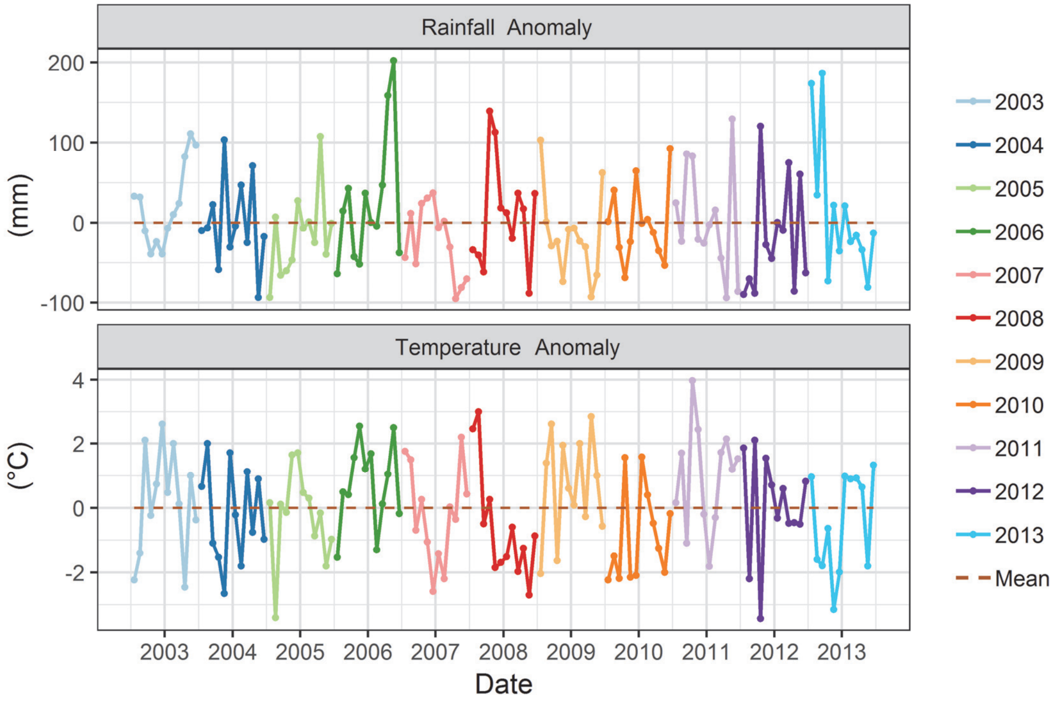

Figure S1. Monthly climate patterns for the study period 2003–2013, recorded at the Puerto de Navacerrada weather station; showing the anomalies (difference from the 2003–2013 mean) for: monthly sum of rainfall (mm) and mean monthly temperature (°C). Compared with longer term conditions, the 2005 summer was the driest since 1947 (37% lower precipitation than the annual summer average) and 1.3°C hotter than annual average for the Iberian Peninsula since 1960 (AEMet, 2015).

Figure S2

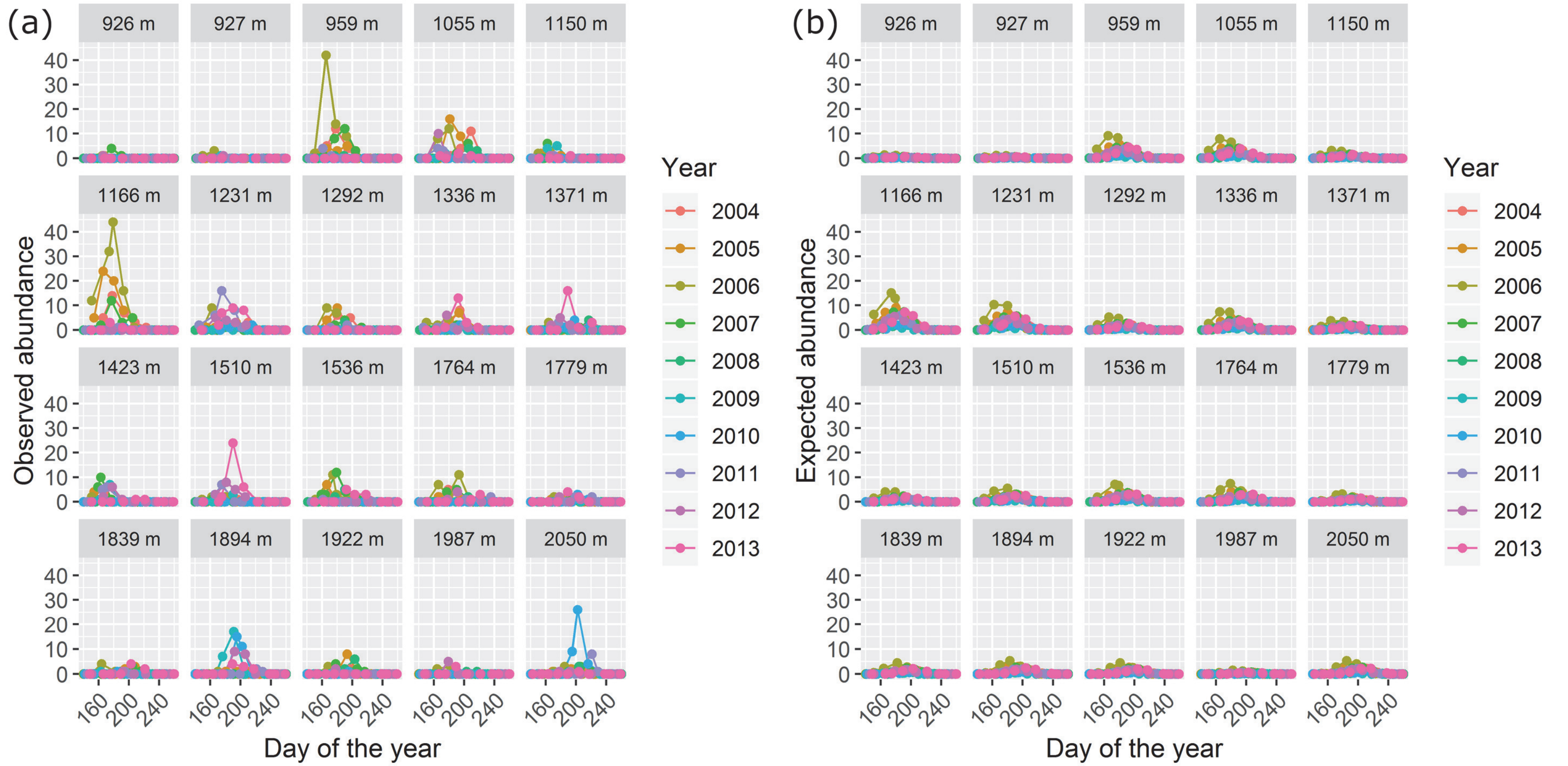

Figure S2. a) Observed abundances and b) expected abundances for *Fabriciana niobe* at 20 sites in the Sierra de Guadarrama, 2004–2013. Expected abundances are those based on the best-fit phenology model parameters, presented in Table 2. The observed and expected abundances correlate well (Spearman's $\rho = 0.525$, $p < 0.001$, n = 2459).

Figure S3

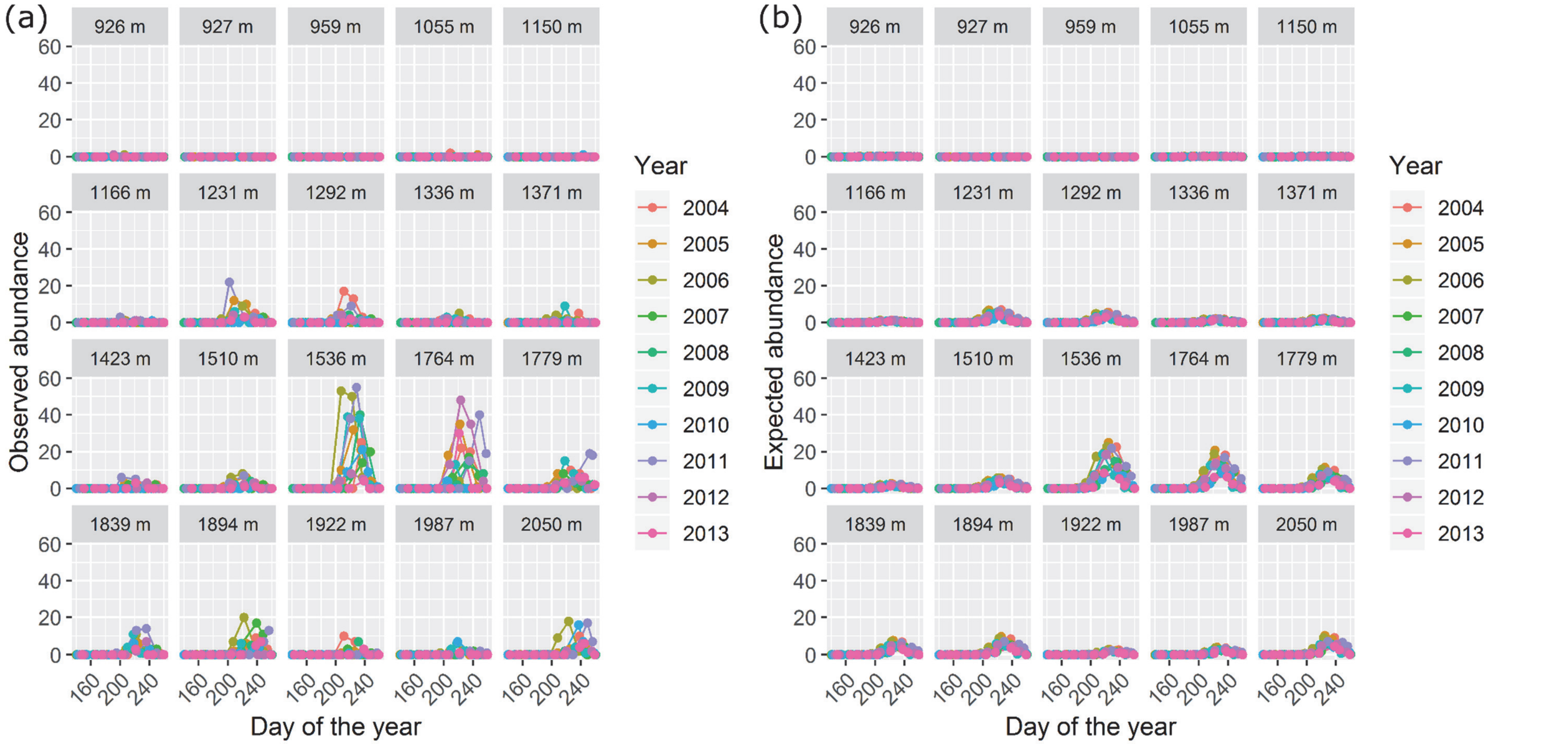

Figure S3. a) Observed abundances and b) expected abundances for *Hesperia comma* at 20 sites in the Sierra de Guadarrama, 2004–2013. Expected abundances are those based on the best-fit phenology model parameters, presented in Table 2. The observed and expected abundances correlate well (Spearman's $\rho = 0.556$, $p < 0.001$, n = 2459).

Figure S4

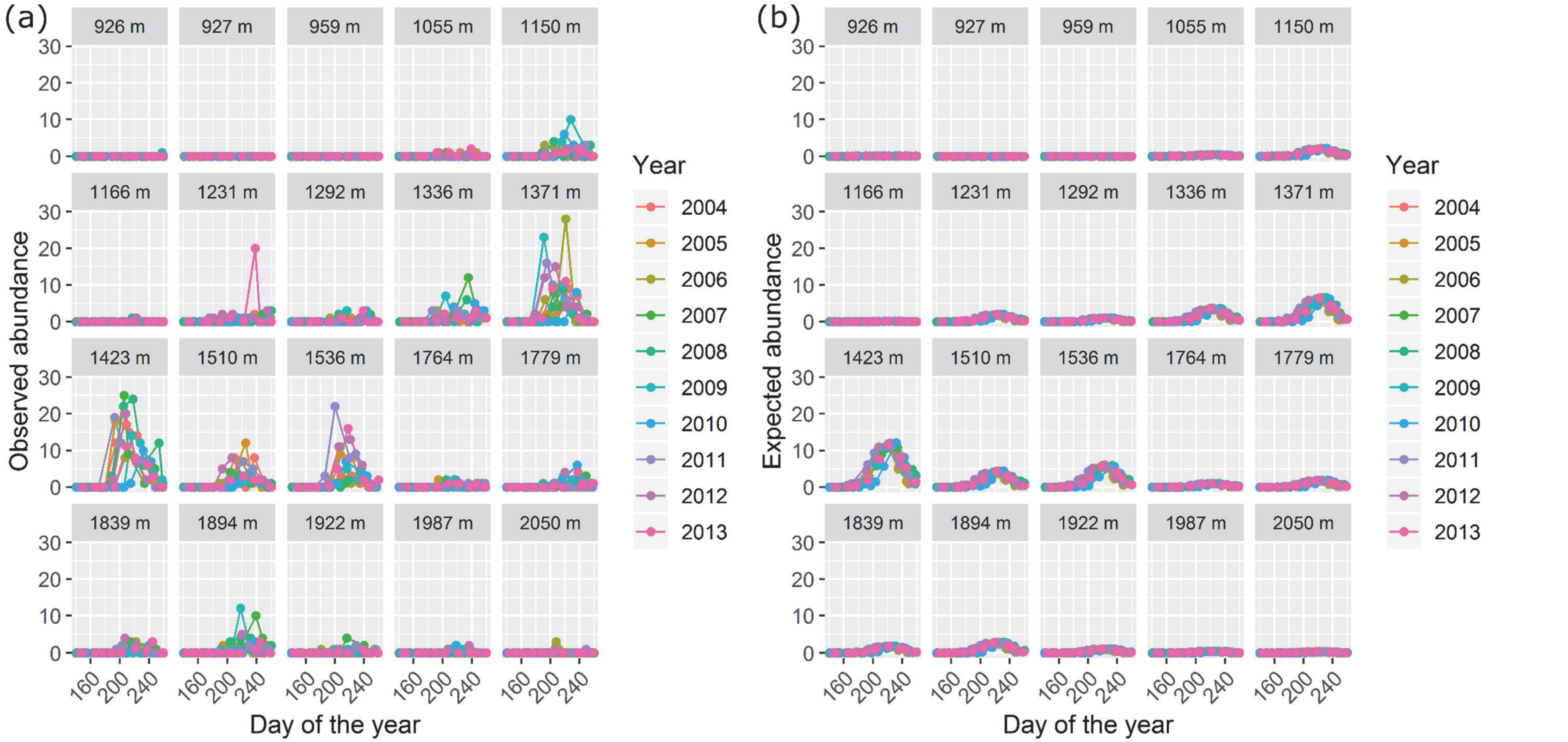

Figure S4. a) Observed abundances and b) expected abundances for *Hipparchia hermione* at 20 sites in the Sierra de Guadarrama, 2004–2013. Expected abundances are those based on the best-fit phenology model parameters, presented in Table 2. The observed and expected abundances correlate well (Spearman's ρ = 0.580, $p < 0.001$, n = 2459).

Figure S5

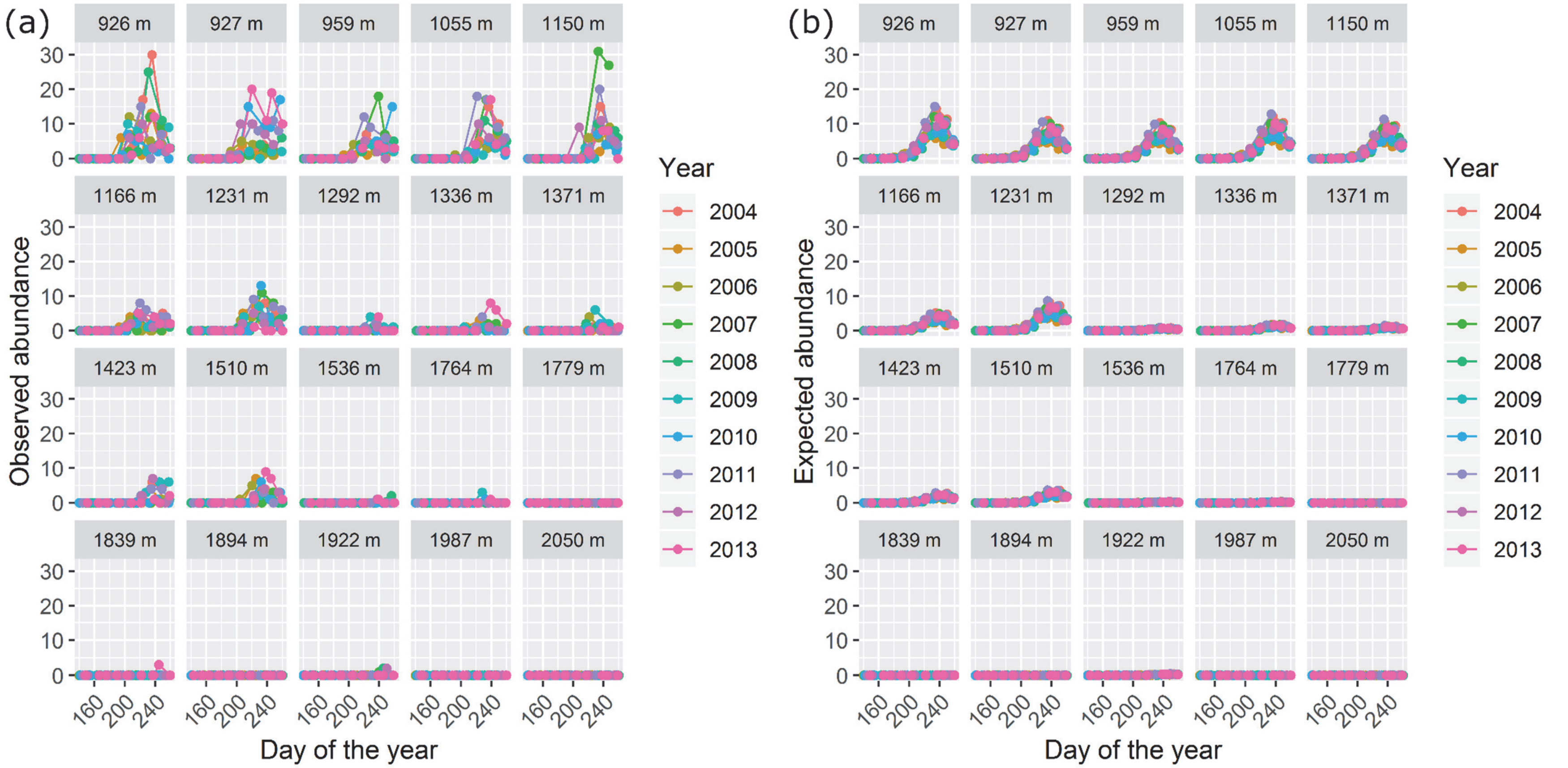

Figure S5. a) Observed abundances and b) expected abundances for *Hipparchia statilinus* at 20 sites in the Sierra de Guadarrama, 2004–2013. Expected abundances are those based on the best-fit phenology model parameters, presented in Table 2. The observed and expected abundances correlate well (Spearman's $\rho = 0.628$, $p < 0.001$, n = 2459).

Figure S6

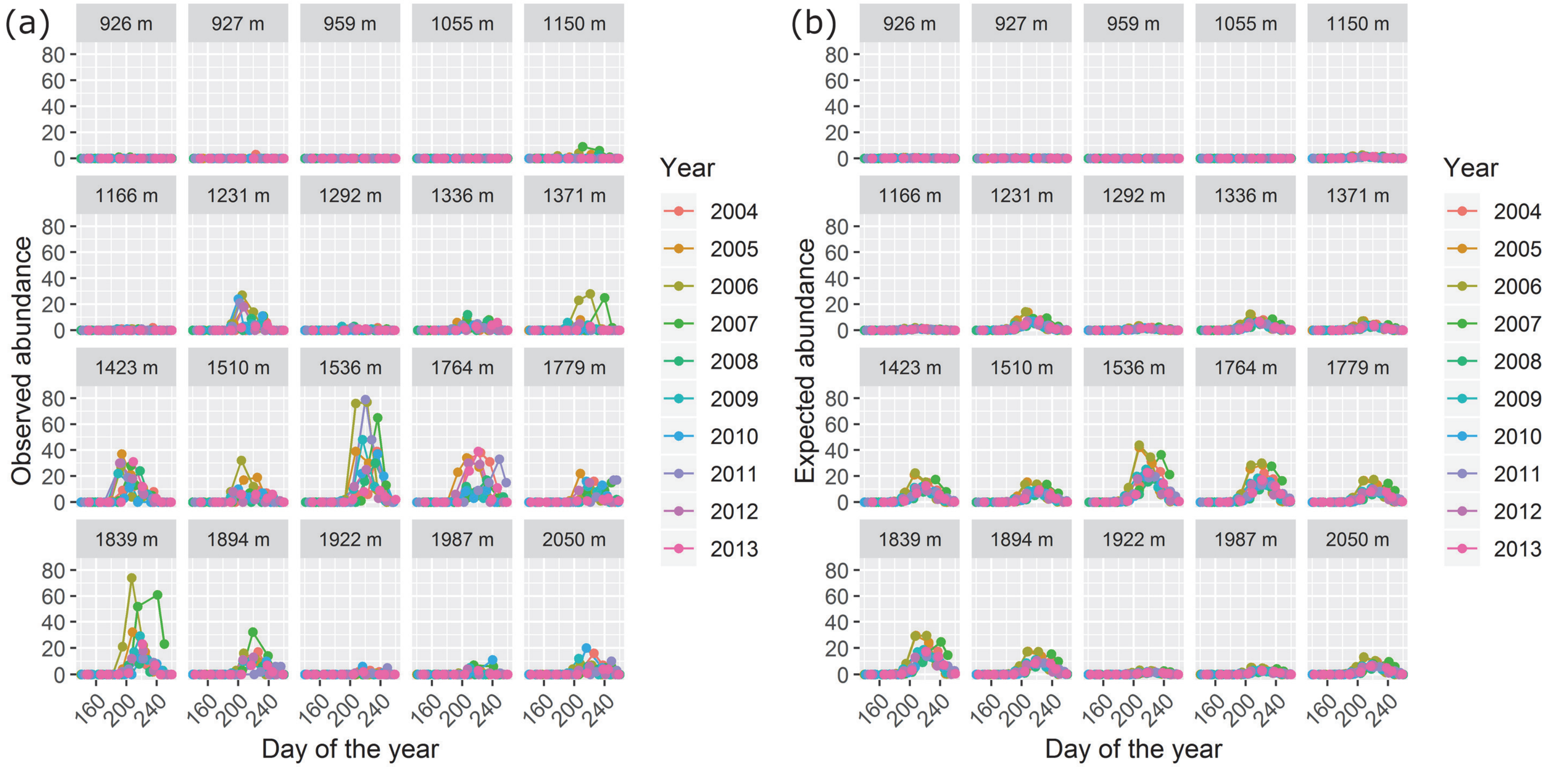

Figure S6. a) Observed abundances and b) expected abundances for *Hyponephele lycaon* at 20 sites in the Sierra de Guadarrama, 2004–2013. Expected abundances are those based on the best-fit phenology model parameters, presented in Table 2. The observed and expected abundances correlate well (Spearman's $\rho = 0.624$, $p < 0.001$, n = 2459).

Figure S7

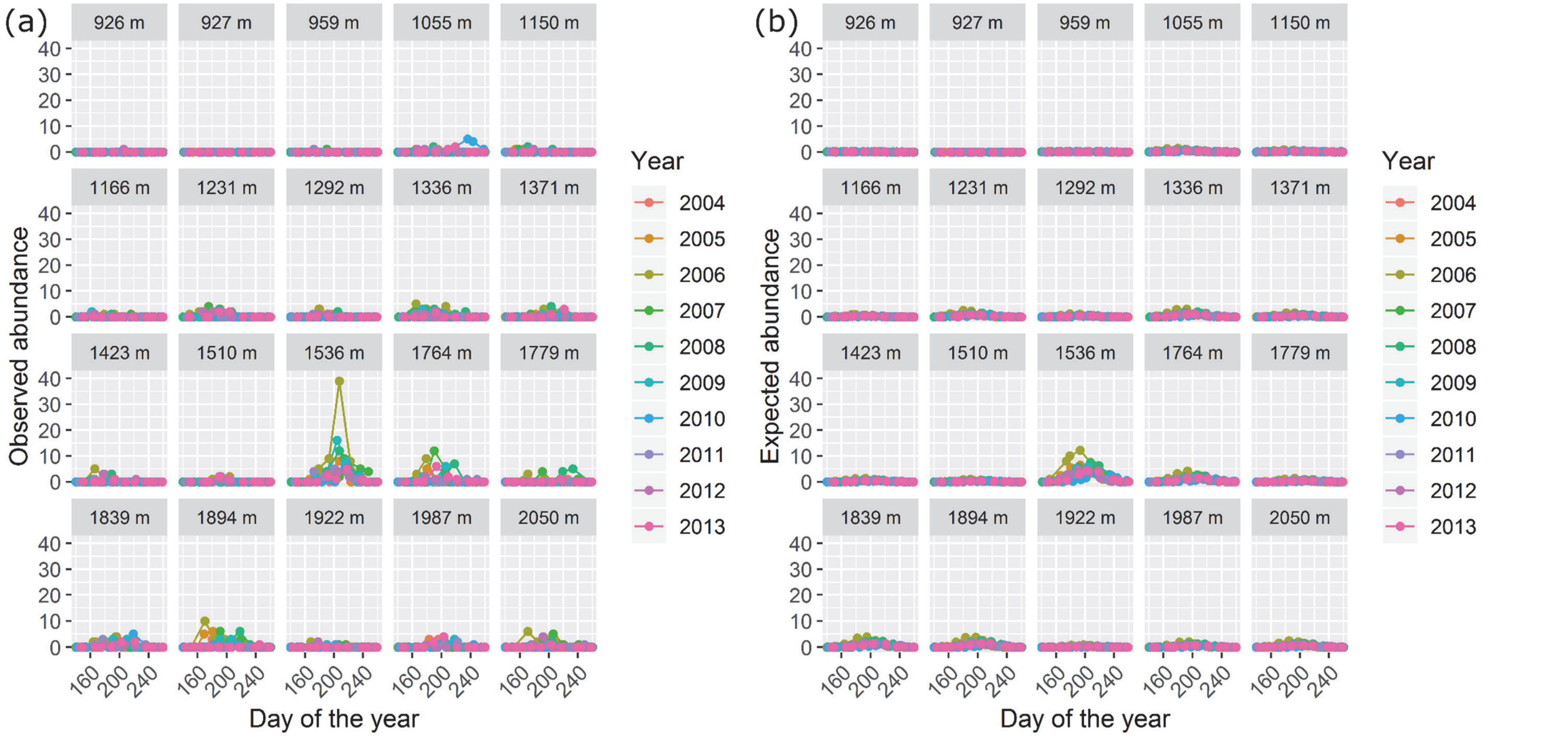

Figure S7. a) Observed abundances and b) expected abundances for *Lycaena alciphron* at 20 sites in the Sierra de Guadarrama, 2004–2013. Expected abundances are those based on the best-fit phenology model parameters, presented in Table 2. The observed and expected abundances correlate well (Spearman's $\rho = 0.471$, $p < 0.001$, n = 2459).

Figure S8

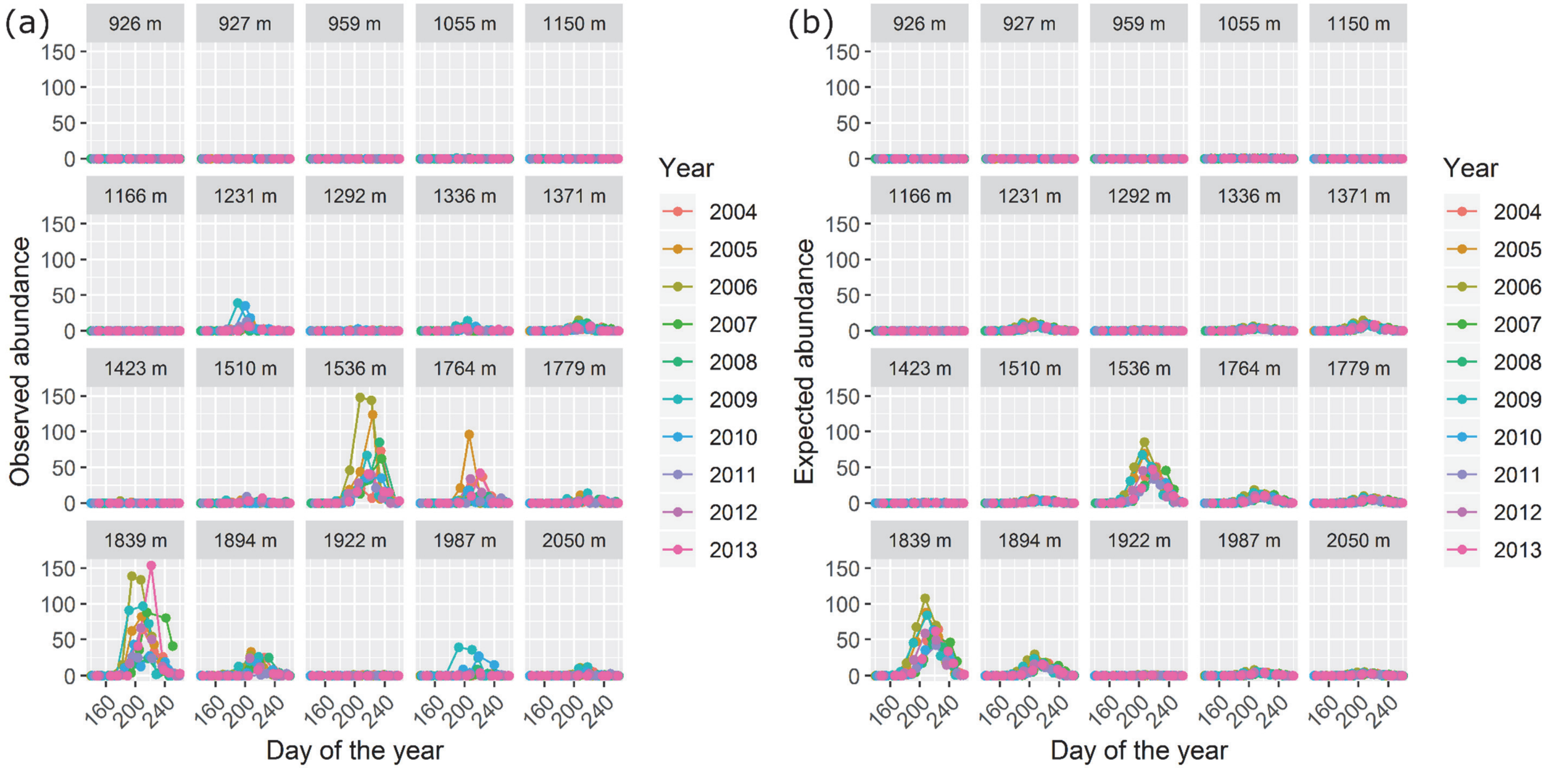

Figure S8. a) Observed abundances and b) expected abundances for *Lycaena virgaureae* at 20 sites in the Sierra de Guadarrama, 2004–2013. Expected abundances are those based on the best-fit phenology model parameters, presented in Table 2. The observed and expected abundances correlate well (Spearman's $\rho = 0.602$, $p < 0.001$, n = 2459).

Figure S9

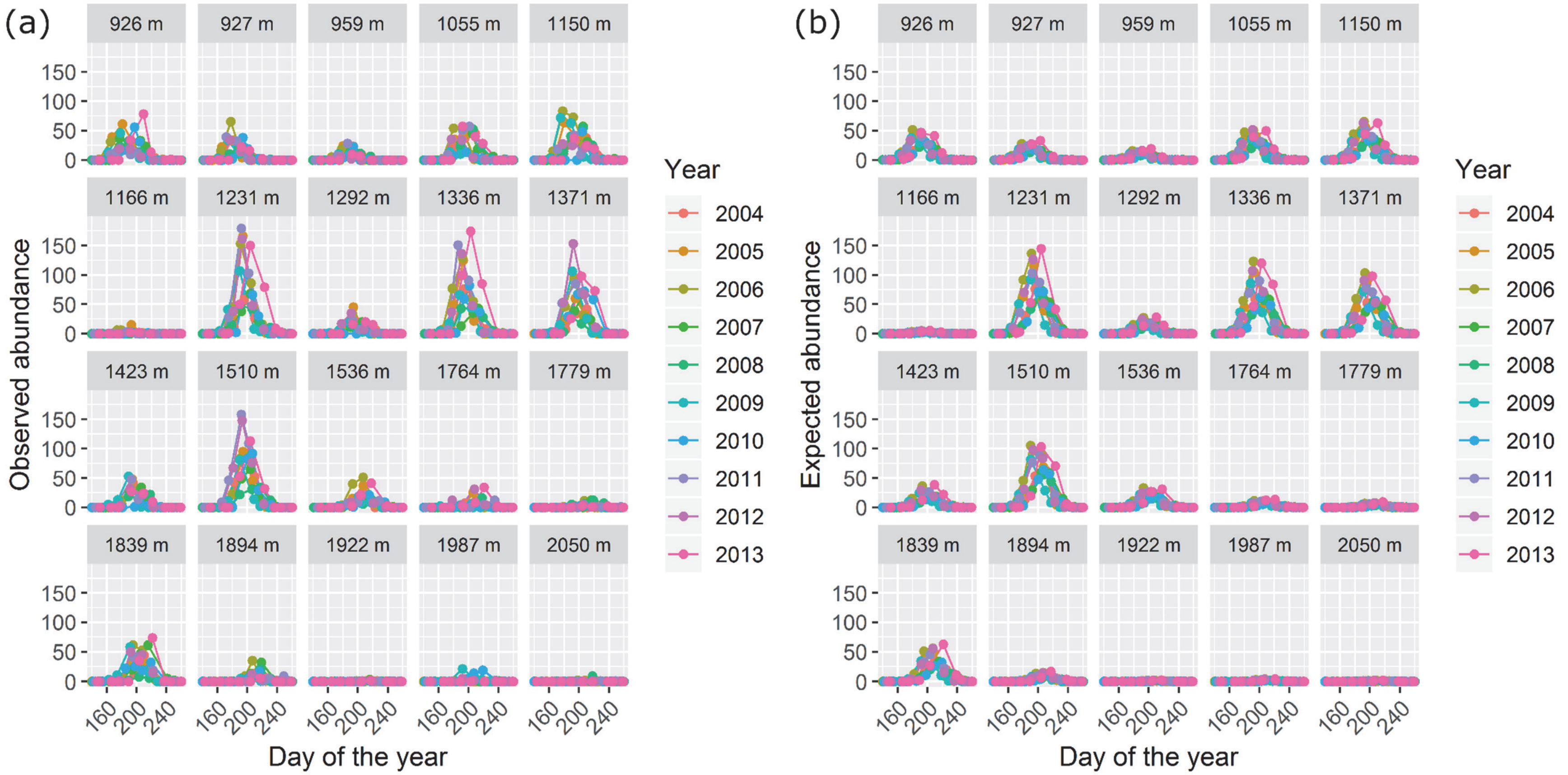

Figure S9. a) Observed abundances and b) expected abundances for *Melanargia lachesis* at 20 sites in the Sierra de Guadarrama, 2004–2013. Expected abundances are those based on the best-fit phenology model parameters, presented in Table 2. The observed and expected abundances correlate well (Spearman's $\rho = 0.748$, $p < 0.001$, $n = 2459$).

Figure S10

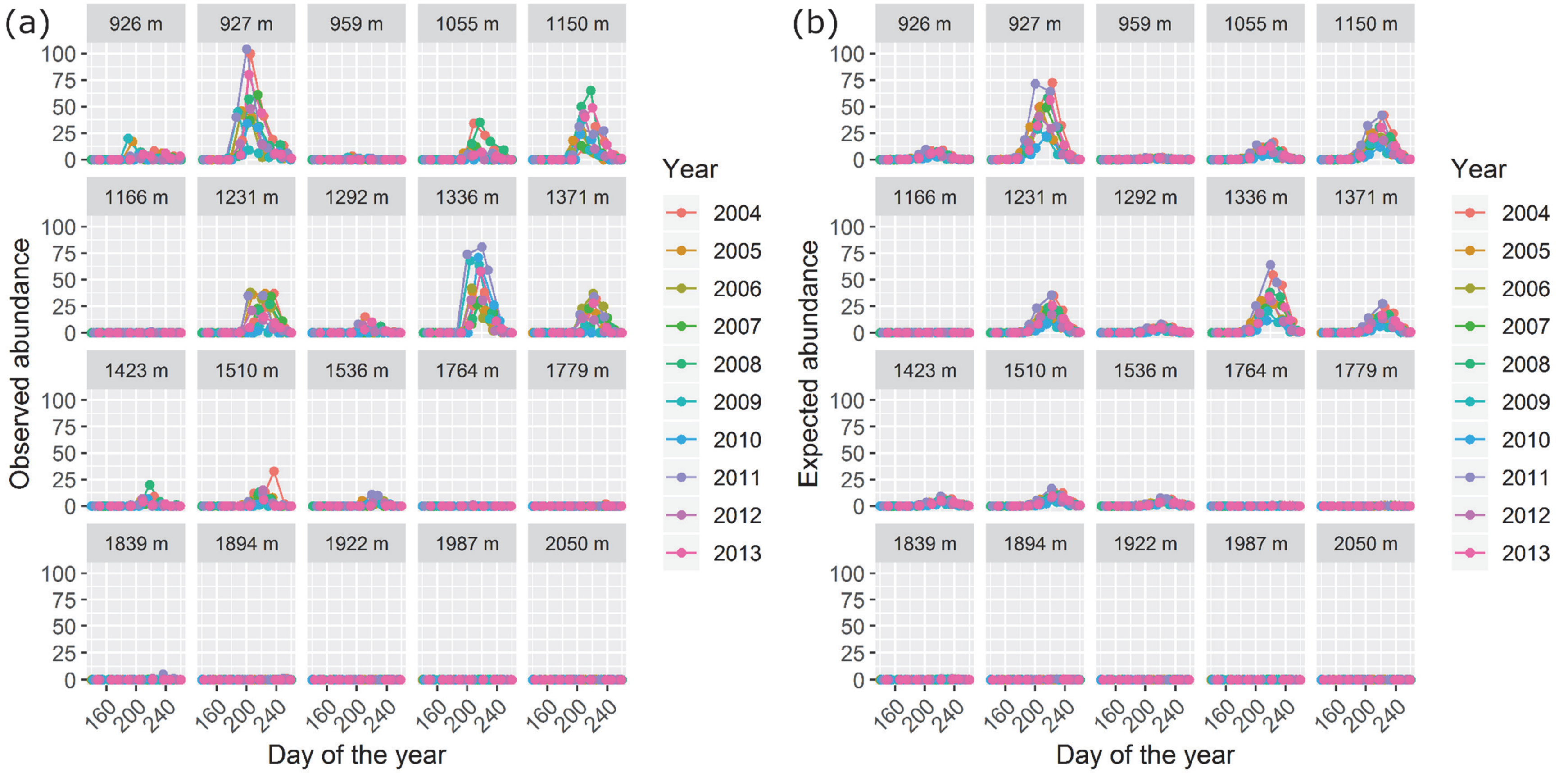

Figure S10. a) Observed abundances and b) expected abundances for *Pyronia tithonus* at 20 sites in the Sierra de Guadarrama, 2004–2013. Expected abundances are those based on the best-fit phenology model parameters, presented in Table 2. The observed and expected abundances correlate well (Spearman's $\rho = 0.592$, $p < 0.001$, n = 2459).

Figure S11

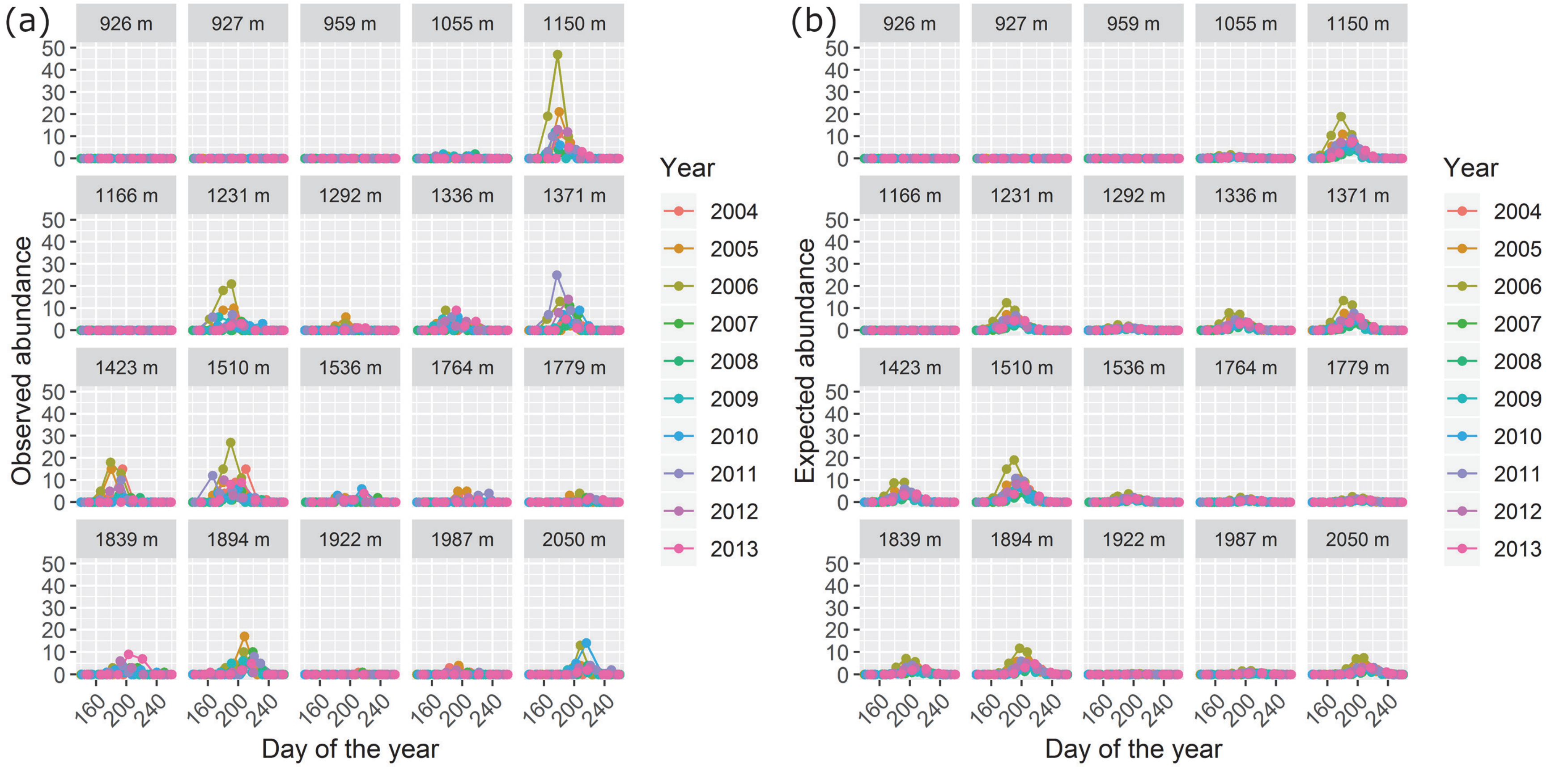

Figure S11. a) Observed abundances and b) expected abundances for *Speyeria aglaja* at 20 sites in the Sierra de Guadarrama, 2004–2013. Expected abundances are those based on the best-fit phenology model parameters, presented in Table 2. The observed and expected abundances correlate well (Spearman's $\rho = 0.515$, $p < 0.001$, n = 2459).

Table S1. Summary of AIC analyses for each species, showing all models with ΔAIC ≤ 6. M_{best} denotes the best AIC model for each species, with subsequent models denoted M_a, M_b,…M_z; the selected 'final' models are denoted †. All models contain parameters 1–4 specified in Table 1 (n_s^*, d^*, s_d, and ϕ; see Table 1 for definitions) and the inclusion of additional parameters is denoted • below. Q_y, R_y and U_y each represent 9 individual parameters (yearly effects). *k* is the number of optimized parameters and *LL* is the log-likelihood of the model.

Species	*Model*	*Model parameters*						*k*	*LL*	ΔAIC
		h_1	h_2	g	Q_y	R_y	U_y			
Fabriciana niobe	M_{best}	•	•		•	•	•	52	-1209.25	0
	M_a†	•	•		•		•	43	-1221.08	5.675
Hesperia comma	M_{best}†	•			•	•	•	51	-1190.16	0
Hipparchia hermione	M_{best}				•	•		41	-1239.13	0
Hipparchia statilinus	M_{best}†	•			•	•	•	51	-1281.06	0
Hyponephele lycaon	M_{best}†	•	•		•	•	•	52	-1592.61	0
Lycaena alciphron	M_{best}†	•	•		•		•	43	-937.68	0
Lycaena virgaureaue	M_{best}†	•	•		•		•	43	-1420.47	0
Melanargia lachesis	M_{best}†	•			•	•	•	51	-2619.93	0
Pyronia tithonus	M_{best}†	•			•		•	42	-1389.59	0
Speyeria aglaja	M_{best}†	•			•		•	42	-1003.08	0

Nested models are shown only if the inclusion of additional parameters improves the AIC value for that model relative to the simpler version.

Table S2. Expected maximum abundance values for all species at all sites (n_i^* in Table 2). Species are indicated as follows, using the initial letters of their Latin bimomial; Fn: *Fabriciana niobe*, Hc: *Hesperia comma*, Hh: *Hipparchia hermione*, Hs: *Hipparchia statilinus*, Hl: *Hyponephele lycaon*, La: *Lycaena alciphron*, Lv: *Lycaena virgaureae*, Ml: *Melanargia lachesis*, Pt: *Pyronia tithonus*, Sa: *Speyeria aglaja*. Note that site elevation does not increase monotonically with site number.

Site	Species									
	Fn	Hc	Hh	Hs	Hl	La	Lv	Ml	Pt	Sa
1	0.5	0.2	0.1	10.0	0.3	0.0	0.0	39.5	6.2	0.0
2	0.4	0.0	0.0	7.7	0.1	0.0	0.0	23.0	48.7	0.0
3	3.5	0.0	0.0	7.3	0.0	0.1	0.0	13.2	1.4	0.0
4	3.0	0.2	0.4	8.6	0.0	0.7	0.3	40.4	10.3	0.7
5	1.2	0.1	2.1	7.6	1.5	0.4	0.0	49.0	25.9	7.5
6	5.5	1.0	0.1	3.8	1.2	0.4	0.0	3.6	0.1	0.0
7	4.4	5.2	2.0	5.9	8.3	1.1	8.3	98.2	21.5	5.0
8	2.0	4.2	1.0	0.6	2.0	0.5	0.8	19.8	4.6	1.0
9	2.9	1.7	3.7	1.4	7.5	1.5	4.1	88.2	34.7	3.5
10	1.5	1.9	6.7	1.0	4.3	0.8	9.2	74.2	15.3	5.6
11	1.6	1.9	12.1	2.0	13.9	0.7	0.4	25.9	4.9	4.1
12	2.1	4.5	4.4	2.6	10.4	0.4	3.9	85.2	8.9	7.6
13	2.5	18.5	6.1	0.2	29.0	5.7	53.6	24.5	4.1	1.6
14	2.6	14.1	1.0	0.2	20.9	1.9	11.7	10.0	0.1	0.8
15	1.1	7.9	1.9	0.0	12.1	0.6	6.3	6.2	0.1	1.0
16	1.6	5.3	1.9	0.1	21.4	1.8	68.1	44.0	0.4	2.9
17	1.9	6.5	2.9	0.0	12.6	1.9	18.6	11.2	0.3	4.7
18	1.6	2.0	1.1	0.3	2.2	0.4	0.8	1.4	0.0	0.2
19	0.5	2.7	0.5	0.0	3.7	1.0	5.0	2.8	0.0	0.7
20	1.9	6.9	0.4	0.0	8.7	1.2	3.4	1.5	0.0	3.1

Table S3. Summary of AIC analyses of rainfall and temperature effects on phenology (Q_y) for all species, showing all models with ΔAIC ≤ 6. M_{best} denotes the best AIC model for each species, with subsequent models denoted M_a, M_b,…M_z; the selected 'final' models are denoted †. *k* is the number of optimized parameters and *LL* is the log-likelihood of the model. Positive and negative effects are denoted + and – respectively, square brackets indicate the parameter with the largest effect size in each model.

Species	Model	Model parameters														
		β_0	R_{1a}	R_{1b}	R_2	R_3	R_4	T_{Jan}	T_{Feb}	T_{Mar}	T_{Apr}	T_{May}	T_{Jun}	*k*	*LL*	ΔAIC
F. niobe	M_{best}	0			–					–		[–]		4	23.58	0.000
	M_a	0								–		[–]	–	4	24.91	2.644
	M_b	0	–									[–]	–	4	25.22	3.264
	M_c	0										[–]	–	3	26.30	3.425
	M_d†	0										[–]		2	28.55	5.942
H. comma	M_{best}	0		+	+								[–]	4	25.12	0.000
	M_a	0			+					–			[–]	4	25.21	0.171
	M_b	0		+						–			[–]	4	25.79	1.331
	M_c	0				–				[–]			–	4	25.84	1.428
	M_d	0								–			[–]	3	26.90	1.559
	M_e	0						+		[–]				3	27.28	2.324
	M_f†	0											[–]	2	28.52	2.790
H. hermione	M_{best}	0		–				–				[–]		4	23.35	0.000
	M_a	0		–								[–]	–	4	23.76	0.825
	M_b	0		–								[–]		3	24.77	0.847
	M_c†	0										[–]		2	26.26	1.836
	M_d	0											[–]	2	27.10	3.516
H. statilinus	M_{best}†	0					+	–				[–]		4	17.52	0.000
H. lycaon	M_{best}†	0		+					+				[–]	4	21.78	0.000
	M_a	0		+						–			[–]	4	23.42	3.280
	M_b	0							+	[–]			–	4	24.45	5.339
	M_c	0			+					–			[–]	4	24.78	5.993
L. alciphron	M_{best}	0	–	+									[–]	4	28.27	9.703
	M_a	0	–			+							[–]	4	28.37	9.902
	M_b	0			–			–				[–]		4	31.10	15.346
	M_c†	0			–							[–]		3	32.20	15.559
L. virgaureae	M_{best}†	0						+			+	[–]		4	22.04	0.000
M. lachesis	M_{best}†	0								[–]	–		–	4	14.77	0.000
P. tithonus	M_{best}†	0									+	[–]		3	18.59	0.000
	M_a	0								–		[–]		3	18.81	0.425
	M_b	0	–									[–]		3	18.99	0.796
	M_c	0		–								[–]		3	19.68	2.170
S. aglaja	M_{best}	0						+			+	[–]		4	19.06	0.000
	M_a†	0						+				[–]		3	22.42	4.721
	M_b	0								[–]	–		–	4	21.69	5.253

M_c	0	–		[–]	–	4	21.71	5.296
M_d	0		+	[–]	–	4	21.79	5.466

Nested models are shown only if the inclusion of additional parameters improves the AIC value for that model relative to the simpler version. All models used Gaussian errors and the identity link function. Combinations of up to three predictors were considered from the following: rain in July–September (R_{1a}) and October–December (R_2) of year y-1, rain in January–March (R_3) and April–June (R_4) of year y and monthly temperatures of January–June in year y (T_{Jan}–T_{Jun}) at the primary weather station, in addition to rain in July–September at the secondary weather station, Colmenar Viejo (R_{1b}).

Table S4. Summary of AIC analyses of rainfall and temperature effects on abundance (ρ_y) for all species, showing all models with ΔAIC ≤ 6. M_{best} denotes the best AIC model for each species, with subsequent models denoted M_a, M_b,…M_z; the selected 'final' models are denoted †. k is the number of optimized parameters and LL is the log-likelihood of the model. Positive and negative effects are denoted + and – respectively, square brackets indicate the parameter with the largest effect size in each model.

**Species**	**Model**	**Model parameters**														
		β_0	R_{1a}	R_{1b}	R_2	R_3	R_4	T_{Jan}	T_{Feb}	T_{Mar}	T_{Apr}	T_{May}	T_{Jun}	k	LL	ΔAIC
F. niobe	M_{best}*	0.95											[–]	2	-9.04	0.000
	M_a†	1.19												1	10.13	0.182
H. comma	M_{best}†*	1.06						–			–		[–]	4	13.30	0.000
H. hermione	–	No yearly abundance effects for *H. hermione*														
H. statilinus	M_{best}	1.00						[+]			+			3	3.21	0.000
	M_a	1.00						[+]	+					3	1.84	2.738
	M_b*	1.05							[–]	+				3	1.47	3.481
	M_c†	1.00												1	-1.58	5.592
H. lycaon	M_{best}†	1.05			+							–	[+]	4	3.34	0.000
L. alciphron	M_{best}†	1.10												1	-5.43	0.000
L. virgaureae	M_{best}*	1.09	+		–		[+]							4	4.83	0.000
	M_a	1.04		–			[–]							3	2.86	1.939
	M_b	1.04				–	[–]							3	1.59	4.489
	M_c†*	0.99											[–]	2	0.26	5.151
M. lachesis	M_{best}	1.06	–				[–]	+						4	4.55	0.000
	M_a	1.06		–			[–]	+						4	3.23	2.638
	M_b	1.06				+	[–]	+						4	3.07	2.952
	M_c*	0.98					[+]	–			–			4	2.88	3.332
	M_d†	1.06												1	-1.39	5.870
P. tithonus	M_{best}†*	1.09		[–]			–							3	4.19	0.000
S. aglaja	M_{best}	1.12					–				+		[+]	4	-3.40	0.000
	M_a	1.12											[+]	2	-5.83	0.856
	M_b	1.12					–			–		[+]		4	-3.83	0.868
	M_c	1.12					–				+	[+]		4	-4.51	2.233
	M_d†	1.12												1	-8.09	3.384

Nested models are shown only if the inclusion of additional parameters improves the AIC value for that model relative to the simpler version. All models used Gaussian errors and the identity link function, except where the use of the inverse link function is denoted *. For models with an inverse link, the sign of the regression coefficients is reversed, such that a negative coefficient reported here is indicative of a positive effect of that variable. Combinations of up to three predictors were considered from the following: rain in July–September (R_{1a}) and October–December (R_2) of year y-1, rain in

January–March (R_3) and April–June (R_4) of year y and monthly temperatures of January–June in year y (T_{Jan}–T_{Jun}) at the primary weather station, in addition to rain in July–September at the secondary weather station, Colmenar Viejo (R_{1b}).

Literature Cited

AEMet (2015). Monthly climatological data available from Agencia Estatal de Meteorología, Ministerio de Agricultura, Alimentacion y Medio-Ambiente, Spanish Government; last accessed via http://www.aemet.es/es/serviciosclimaticos/vigilancia_clima/resumenes April 2019.
