# Supplementary material for: Linking inter‐annual variation in environment, phenology, and abundance for a montane butterfly community
Source: Ecology. 2019 Nov 29;101(1):e02906. doi: 10.1002/ecy.2906 (PMC9285533; doi:10.1002/ecy.2906)
Supplement: Supplementary file 4 [file ECY-101-e02906-s001.pdf]

Stewart, J.E., Gutiérrez Illán, J., Richards, S.A., Gutiérrez, D. and Wilson, R.J. 2019. Linking inter-annual variation in environment, phenology, and abundance for a montane butterfly community. *Ecology*.

Data S1

R script detailing the phenology model described and used in Stewart et al. (2019): Linking inter-annual variation in environment, phenology, and abundance for a montane butterfly community.

Authors

James E. Stewart
College of Life and Environmental Sciences, University of Exeter
Exeter EX4 4PS United Kingdom
j.e.stewart8@gmail.com

Javier Gutiérrez Illán
Department of Entomology, Washington State University
Pullman, Washington 99164-6382 USA

Shane A. Richards
School of Natural Sciences, University of Tasmania
Hobart, Tasmania 7001, Australia

David Gutiérrez
Área de Biodiversidad y Conservación, Universidad Rey Juan Carlos
Móstoles, Madrid E28933, Spain

Robert J. Wilson
Departamento de Biogeografía y Cambio Global, Museo Nacional de Ciencias Naturales (MNCN-CSIC),
Madrid E28006, Spain

File list (files found within DataS1.zip)

`PhenologyModelRCode.R`

Description

This R (R Core Team, 2017) file outlines the R code for the phenology model described in our manuscript. All code is described and explained using comments within the R file. These comments also refer back to our manuscript to improve clarity and ease of use. All code can be run in R version 3.4.1 or later, using the R packages detailed in the code on lines 19–26.

R Core Team (2017). R: A language and environment for statistical computing. R Foundation for Statistical Computing, Vienna, Austria. www.r-project.org
